# GH20 and GH84 β-*N*-acetylglucosaminidases with different linkage specificities underpin mucin *O*-glycan breakdown capability of *Bifidobacterium bifidum*

**DOI:** 10.1016/j.jbc.2023.104781

**Published:** 2023-05-03

**Authors:** Hiromi Takada, Toshihiko Katoh, Mikiyasu Sakanaka, Toshitaka Odamaki, Takane Katayama

**Affiliations:** 1 Graduate School of Biostudies, Kyoto University, Sakyo-Ku, Kyoto, Japan; 2 Next Generation Science Institute, Morinaga Milk Industry Co Ltd, Zama, Kanagawa, Japan

**Keywords:** β-*N*-acetylglucosaminidase, *Bifidobacterium bifidum*, glycoside hydrolase, mucin, mucin *O*-glycan, mass spectrometry, substrate specificity

## Abstract

Intestinal mucous layers mediate symbiosis and dysbiosis of host–microbe interactions. These interactions are influenced by the mucin *O*-glycan degrading ability of several gut microbes. The identities and prevalence of many glycoside hydrolases (GHs) involved in microbial mucin *O*-glycan breakdown have been previously reported; however, the exact mechanisms and extent to which these GHs are dedicated to mucin *O*-glycan degradation pathways warrant further research. Here, using *Bifidobacterium bifidum* as a model mucinolytic bacterium, we revealed that two β-*N*-acetylglucosaminidases belonging to the GH20 (BbhI) and GH84 (BbhIV) families play important roles in mucin *O*-glycan degradation. Using substrate specificity analysis of natural oligosaccharides and *O*-glycomic analysis of porcine gastric mucin (PGM) incubated with purified enzymes or *B. bifidum* carrying *bbhI* and/or *bbhIV* mutations, we showed that BbhI and BbhIV are highly specific for β-(1→3)- and β-(1→6)-GlcNAc linkages of mucin core structures, respectively. Interestingly, we found that efficient hydrolysis of the β-(1→3)-linkage by BbhI of the mucin core 4 structure [GlcNAcβ1-3(GlcNAcβ1-6)GalNAcα-*O*-Thr] required prior removal of the β-(1→6)-GlcNAc linkage by BbhIV. Consistent with this, inactivation of *bbhIV* markedly decreased the ability of *B. bifidum* to release GlcNAc from PGM. When combined with a *bbhI* mutation, we observed that the growth of the strain on PGM was reduced. Finally, phylogenetic analysis suggests that GH84 members may have gained diversified functions through microbe–microbe and host–microbe horizontal gene transfer events. Taken together, these data strongly suggest the involvement of GH84 family members in host glycan breakdown.

The gastrointestinal tract is covered by mucous layers that are mainly composed of mucin glycoproteins secreted from intestinal epithelial cells (1, 2). The glycoproteins are heavily modified at the Ser and Thr residues with carbohydrate chains (mucin *O*-glycans) whose abundance is estimated to be more than 50% of the molecular weight of the mucins. Four major core *O*-GalNAc glycan structures are present in the human gastrointestinal tract. Among them, *O*-glycans with the core 3 structure (GlcNAcβ1-3GalNAcα-*O*-Ser/Thr) are widely distributed along the intestine, whereas core 2 [Galβ1-3(GlcNAcβ1-6)GalNAcα-*O*-Ser/Thr]- and core 4 [GlcNAcβ1-3(GlcNAcβ1-6)GalNAcα-*O*-Ser/Thr]-carrying *O*-glycans are found in the distal colon and ileum, respectively (3).

Mucin *O*-glycans are important not only for maintaining the gel-forming structure of the mucous layers (4, 5, 6) but also for preventing pathogens and toxins from adhering to intestinal cells by acting as decoys of sugar receptors (7, 8). In addition, recent studies showed that certain mucin *O*-glycan structures could suppress the growth of harmful microbes (9, 10). Inactivation of *cosmc*, a chaperon of the enzyme synthesizing the mucin core 1 structure (T-antigen: Galβ1-3GalNAcα*-O*-Ser/Thr), causes spontaneous intestinal inflammation in conventional mice due to the loss of mucous layer integrity (11). These results indicate the importance of mucin *O*-glycans in regulating the gut microbiota. On the other hand, mucin *O*-glycans also serve as a carbon source for several bacterial species in the gut (12, 13). A mutant *Bacteroides thetaiotaomicron* deficient in mucin *O*-glycan utilization showed decreased fitness in the mouse gut ecosystem (14). Desai *et al.* reported that gut bacterial genes that can degrade host glycans, including mucin, were upregulated in mice fed fiber-free diets (15). Mucins are thus the frontline in mediating the interactions between host and gut microbes (13, 16). Therefore, understanding how gut microbes degrade mucin *O*-glycans is pivotal to gaining mechanistic insights into the formation and persistence of gut microbiota, which can eventually influence host health and diseases (17).

*Bifidobacterium bifidum* colonizes the guts of a wide range of animals (18). The high prevalence of the species in various mammalian guts has been attributed to its mucin *O*-glycan degrading capability demonstrated *in vitro*. Quite recently, an *in vivo* mucin *O*-glycan degrading ability of the species was also demonstrated using a gnotobiotic mouse model (19). *B. bifidum* commonly possesses a set of surface-anchored extracellular glycoside hydrolases (GHs) that degrade mucin *O*-glycans (20). The enzymes characterized so far are 1,2-α-l-fucosidase specific for H-antigen (21, 22), 1,3/4-α-l-fucosidase specific for Lewis antigens (23, 24), two sialidases (25, 26), lacto-*N*-biosidase specific for type-1 chains (Galβ1-3GlcNAcβ-*O*-R) (27, 28), β-1,4-galactosidase (29), β-1,3-*N*-acetylglucosaminidase (29), sulfoglycosidase releasing 6-*O*-sulfo-β-*N*-acetylglucosamine (GlcNAc6S) (19, 30), α-galactosidase specific for blood group substance B (31), and α-*N*-acetylglucosaminidase specific for a terminal GlcNAcα1-4Gal linkage (32). The presence of a homolog of endo-α-*N*-acetylgalactosaminidase that releases galacto-*N*-biose (Galβ1-3GalNAc) from T antigen has also been reported (33). The specificities of these GHs can mostly explain the mucin *O*-glycan degradation behavior of *B. bifidum* that was observed when the bacterium was grown on porcine gastric mucin (PGM) as the carbon source and the glycoprotein was subsequently subjected to *O*-glycomic analysis (20). However, there is an enzyme yet to be characterized in order to fully understand the pathway of mucin *O*-glycan degradation, *i.e.*, a β-*N*-acetylglucosaminidase(s) hydrolyzing β-(1→6)-linkages present in mucin core 2 and core 4 structures remained to be identified.

The activity of microbial β-*N*-acetylglucosaminidases that act on β-(1→6)-linkages has not been well described to date. In the limited studies available, Pluvinage *et al.* showed by a thin-layer chromatography analysis that four GH84 paralogs of *Clostridium perfringens* release GlcNAc from the mucin core-2 oligosaccharide with their promiscuous linkage specificities (34). Garrido *et al.* showed that a GH20 β-*N*-acetylglucosaminidase of *Bifidobacterium longum* subsp. *infantis* (Blon_0732) acts on both β-(1→3)- and β-(1→6)-linkages found in human milk oligosaccharides (HMOs) (35). In both cases, oligosaccharides were used as a substrate, and therefore, activity on *O*-glycans attached to intact glycoproteins has not been addressed. Here, through enzymatic, genetic, and *O*-glycomic analyses, we show that *B. bifidum* utilizes two distinct β-*N*-acetylglucosaminidases belonging to GH20 and GH84 for efficiently degrading and assimilating mucin *O*-glycans. Our study strongly suggests the involvement of bacterial GH84 members in host glycan breakdown, in addition to the known regulation of signal transduction in higher eukaryotes *via* the removal of protein *O*-GlcNAc (36, 37).

## Results

### Search for β-*N*-acetylglucosaminidases involved in mucin *O*-glycan breakdown

**Figure 1:**
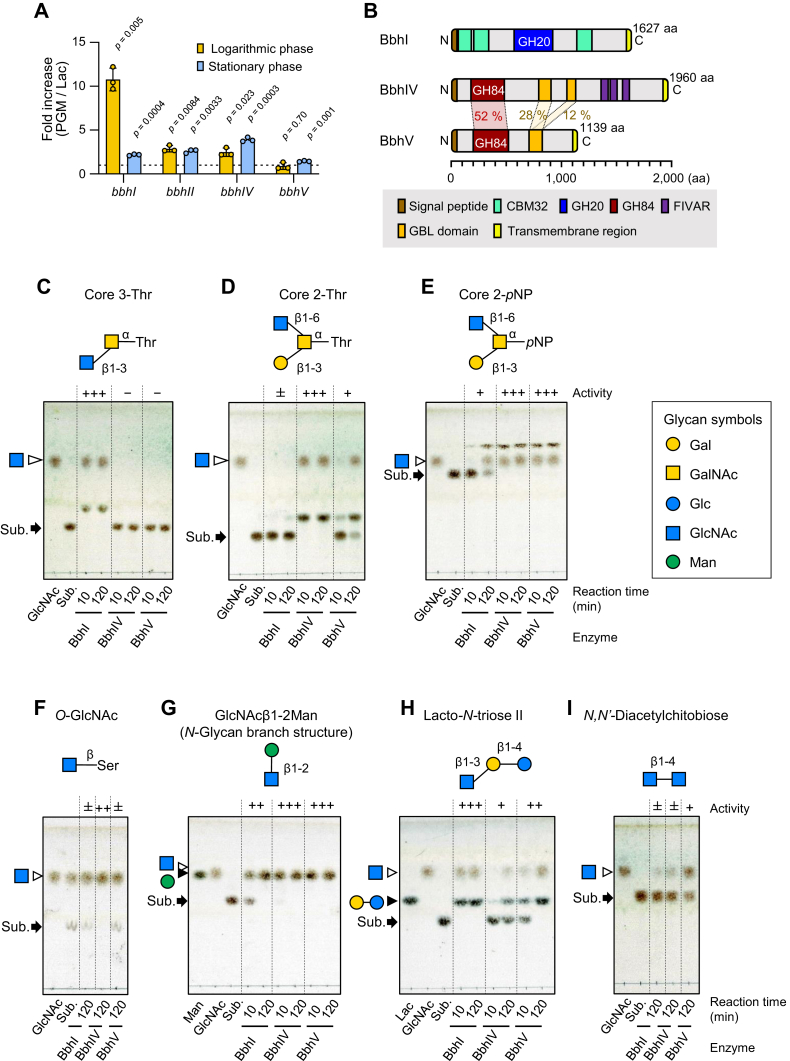
**Characterization of BbhI (GH20), BbhIV (GH84), and BbhV (GH84) of *B. bifidum* JCM 1254.***A*, fold increase of the *bbhI*, *bbhIV*, and *bbhV* transcripts in the cells grown on PGM compared with those grown on Lac. *B. bifidum* JCM 1254 was cultivated in modified de Man-Rogosa-Sharpe medium supplemented with 0.5% porcine gastric mucin (PGM) or Lac as a sole carbon source to the logarithmic growth phase (*A*_600_ = 0.4 ∼ 0.6) and stationary phase (*A*_600_ = 0.8 ∼ 3.0). The relative expression level of each gene was determined with *uvrD* as a reference gene. The bars and whiskers represent mean ± SD of three independent experiments. Student *t* test was used for statistical analysis, and *p* values obtained are shown. *B*, schematic representation of the domain structures of BbhI, BbhIV, and BbhV. A signal peptide, carbohydrate-binding module 32 (CBM32), glycoside hydrolase family 20 (GH20) domain, glycoside hydrolase family 84 (GH84) domain, the found in various architectures (FIVAR) domain, galactose-binding-like (GBL) domain, and transmembrane region are shown in *brown*, *pale green*, *dark blue*, *wine red*, *purple*, *orange*, and *yellow*, respectively. Signal peptides and transmembrane regions were predicted by the SOSUI (39) and TMHMM-2.0 (73) servers, respectively. Domain architecture was analyzed by the Protein families database (Pfam) (70). *C*–*I*, TLC analysis of the reaction products. Purified recombinant BbhI, BbhIV, and BbhV (1 μM each) were incubated with 4 mM of mucin core 3-Thr (*C*), core 2-Thr (*D*), core-2 *p*NP (*E*), *O*-GlcNAc (*F*), GlcNAcβ1-2Man (*G*), lacto-*N*-triose II (*H*), and *N*, *N*′-diacetylchitobiose (*I*). The reactions were stopped at 10 min and 120 min by heating at 95 °C. *Arrows* indicate substrates. GlcNAc, Man, and Lac were used as standard sugars. See Table 1 footnote for activity evaluation expressed by +/−. Glycan symbols are depicted according to the nomenclature committee of the Consortium for Functional Glycomics (72).

β-*N*-Acetylglucosaminidases and β-*N*-acetylhexosaminidases have been found in GH3, 5, 18, 20, 84, 85, 109, 116, and 163 (38). The genomes of *B. bifidum* available in the CAZy genome database commonly encode one GH3, four GH20, and two GH 84 enzymes, and among the GH members, three GH20 and two GH84 enzymes possess signal peptides at the N termini as revealed by the SOSUI server (39), while others are presumed to be located intracellularly. The three extracellular GH20 enzymes have been identified to be lacto-*N*-biosidase (LnbB), β-1,3-*N*-acetylglucosaminidase (BbhI) (29), and 6-*O*-sulfo-β-*N*-acetylglucosaminidase (sulfoglycosidase) (BbhII) (30) as mentioned above, whereas two GH84 enzymes have not been characterized yet. To search for possible β-*N*-acetylglucosaminidases involved in mucin *O*-glycan breakdown, we first examined transcription profiles of BbhI (GH20) and two GH84 genes. Sulfoglycosidase BbhII (GH20) was also included in the analysis, as its transcription was previously shown to be upregulated when *B. bifidum* is grown in the medium supplemented with PGM (30). *B. bifidum* JCM 1254, whose genome was recently determined in our laboratory (40), was grown in modified de Man-Rogosa-Sharpe (mMRS) medium supplemented with PGM as a carbon source. Lactose (Lac)-supplemented medium was used for comparison. The two GH84 enzymes, *i.e.*, locus tags BbifJCM1254_12430 and BbifJCM1254_17960, were named BbhIV (GenBank accession GJM46876.1) and BbhV (GenBank accession GJM47429.1), respectively. Cultivation of the strain in a PGM-supplemented medium resulted in the upregulation of *bbhI*, *bbhII*, and *bbhIV* transcription at the logarithmic growth phase by 11-, 2.9-, and 2.5-fold, respectively, as compared with cells grown on Lac (Fig. 1*A*, *t* test). At the stationary phase, the transcription levels of *bbhI*, *bbhII*, and *bbhIV* were still higher for the cells grown on PGM than for those grown on Lac, with the *bbhIV* transcription level being the highest (3.9-fold). *bbhV* expression was not induced at the exponential phase but was slightly upregulated at the stationary phase with statistical significance. As a crystallographic study has recently shown that sulfoglycosidase BbhII (GH20) is highly specific for 6-*O*-sulfo-GlcNAc (19), we assumed BbhI (GH20) and BbhIV (GH84) to be β-*N*-acetylglucosaminidases involved in mucin *O*-glycan breakdown and thus chose them for further analysis. BbhV (GH84) was also included in the analysis as little is known about bacterial GH84 members.

### Characterization of BbhI, BbhIV, and BbhV of *B. bifidum*

The domain structures of BbhI, BbhIV, and BbhV are shown in Figure 1*B*. BbhI contains a GH20 catalytic domain and three CBM32 domains (29). BbhIV is a 1960-amino-acid (aa) polypeptide with a signal sequence (1–29 aa), a GH84 catalytic domain (184–486 aa), two galactose-binding-like (GBL) domains (820–931 and 1075–1154 aa), three found in various architectures (FIVAR) domains (1364–1425, 1432–1496, and 1567–1625 aa), and a C-terminal transmembrane region (1935–1954 aa). BbhV has a similar domain architecture but lacks FIVAR domains. It consists of an N-terminal signal peptide (1–34 aa), a GH84 catalytic domain (212–524 aa), a GBL domain (727–848 aa), and a C-terminal transmembrane region (1112–1134 aa) in its 1139-aa polypeptide sequence. The amino acid identity of the GH84 catalytic domain between BbhIV and BbhV is 52%, while the identities of the GBL domain of BbhV are 28% and 12% with those of the upstream and the downstream regions of BbhIV, respectively. The two GBL domains within BbhIV share only 22% identity. For characterization, BbhIV (29–1934 aa residues) and BbhV (35–1111 aa residues) were recombinantly expressed as C-terminal hexahistidine (His_6_)-tagged proteins. BbhI (33–1604 aa residues) was expressed as described previously (29). Molecular sizes of purified BbhIV and BbhV proteins were estimated to be 264 and 171 kDa by size-exclusion chromatography, suggesting a monomer and a dimer formation in solution, respectively (Supporting Information Fig. S1, *A* and *B*). BbhIV and BbhV showed the maximum activity at pH 5.0 and 5.5 for GlcNAcβ-*p*NP and were stable in a pH range of 5.5 to 8.0 and 7.5 to 8.0, respectively. Both enzymes did not lose activity under 45 °C. The *K*_m_ and *k*_cat_ values for the substrate were 1.6 mM and 67 s^−1^ for BbhIV and 1.0 mM and 73 s^−1^ for BbhV (Supporting Information Fig. S1*C*), which are comparable with those reported for BbhI (1.2 mM and 210 s^−1^ at pH 6.0) (29).

Substrate specificities of BbhI, BbhIV, and BbhV toward β-linked GlcNAc residues were determined using several natural and chromogenic substrates, and the results are summarized in Table 1. BbhI efficiently hydrolyzed a β-(1→3)-GlcNAc linkage in the mucin core 3 structure, while it hardly acted on a β-(1→6)-GlcNAc linkage in the mucin core 2 structure (Fig. 1, *C* and *D*). On the other hand, BbhIV and BbhV were inactive on a core 3-GlcNAc linkage but were active on a core 2-GlcNAc linkage. BbhIV showed considerably higher activity than BbhV on the core 2 structure when the reducing-end sugar is linked with Thr (core 2-Thr). However, both enzymes showed high activity on the same linkage when the trisaccharide is modified with *p*NP at the reducing end (Fig. 1*E*). Apart from mucin *O*-glycan substrates, GlcNAcβ-*O*-Ser (protein *O*-GlcNAc), which is formed by posttranslational protein modification in higher eukaryotes, was hardly hydrolyzed by BbhI and BbhV but hydrolyzed by BbhIV (Fig. 1*F*). The disaccharide GlcNAcβ1-2Man found in *N*-glycans was efficiently hydrolyzed by BbhIV and BbhV but was not readily hydrolyzed by BbhI (Fig. 1*G*). The rate of hydrolysis of a β-(1→3)-GlcNAc linkage in lacto-*N*-triose II differed among the three enzymes, and it did not accord with the results observed for the β-(1→3)-GlcNAc linkage present in the mucin core 3-Thr structure (Fig. 1*C* *versus* 1*H*), indicating that BbhIV and BbhV differentially recognize the substrates at their (+) subsite(s). *N*,*N′*-Diacetylchitobiose (GlcNAcβ1-4GlcNAc) served as a poor substrate for the three enzymes (Fig. 1*I*). Both BbhIV and BbhV showed a high specificity at their (−) subsite. The activity of BbhIV for GalNAcβ-*p*NP was 0.3% of its GlcNAcβ-*p*NP-hydrolyzing activity, while BbhV was inactive on GalNAcβ-*p*NP. None of the three enzymes hydrolyzed GlcNAc6Sβ-*p*NP. Note that both GH20 and GH84 adopt the substrate-assisted mechanism for catalysis (36, 41).

**Table 1:** Summary of the substrate specificities of BbhI, BbhIV, and BbhV of *B. bifidum*

Substrate^a^	Structure	Activity^b^		
		BbhI	BbhIV	BbhV
*N*-Glycan branching structure	GlcNAcβ1-2Man	++	+++	+++
Lacto-*N*-triose II	GlcNAcβ1-3Galβ1-4Glc	+++	+	++
*N*,*N′*-Diacetylchitobiose	GlcNAcβ1-4GlcNAc	±	±	+
*O*-GlcNAc	GlcNAcβ-*O*-Ser	±	++	±
Core 2-*p*NP	Galβ1-3(GlcNAcβ1-6)GalNAcα-*p*NP	+	+++	+++
Core 2-Thr	Galβ1-3(GlcNAcβ1-6)GalNAcα-*O-*Thr	±	+++	+
Core 3-Thr	GlcNAcβ1-3GalNAcα-*O-*Thr	+++	−	−
Core 4-Thr	GlcNAcβ1-3(GlcNAcβ1-6) GalNAcα-*O-*Thr	+	+++	++

a Activities of BbhIV and BbhV for GlcNAcβ-*p*NP and GalNAcβ-*p*NP are described in the text. Activity of BbhI for GalNAcβ-*p*NP is 0.3% of that for GlcNAcβ-*p*NP (29).

b Activities are expressed as follows. (+++), complete hydrolysis within 10 min; (++), complete hydrolysis within 10 to 120 min; (+), partial hydrolysis within 120 min; (±), marginal hydrolysis within 120 min; (−), no hydrolysis within 120 min. The substrates and enzymes were used at the final concentrations of 4 mM and 1 μM, respectively. See Figure 1*C*‒I and 5A.

### *O-*Glycan analysis of PGM treated with BbhI, BbhIV, and BbhV

**Figure 2:**
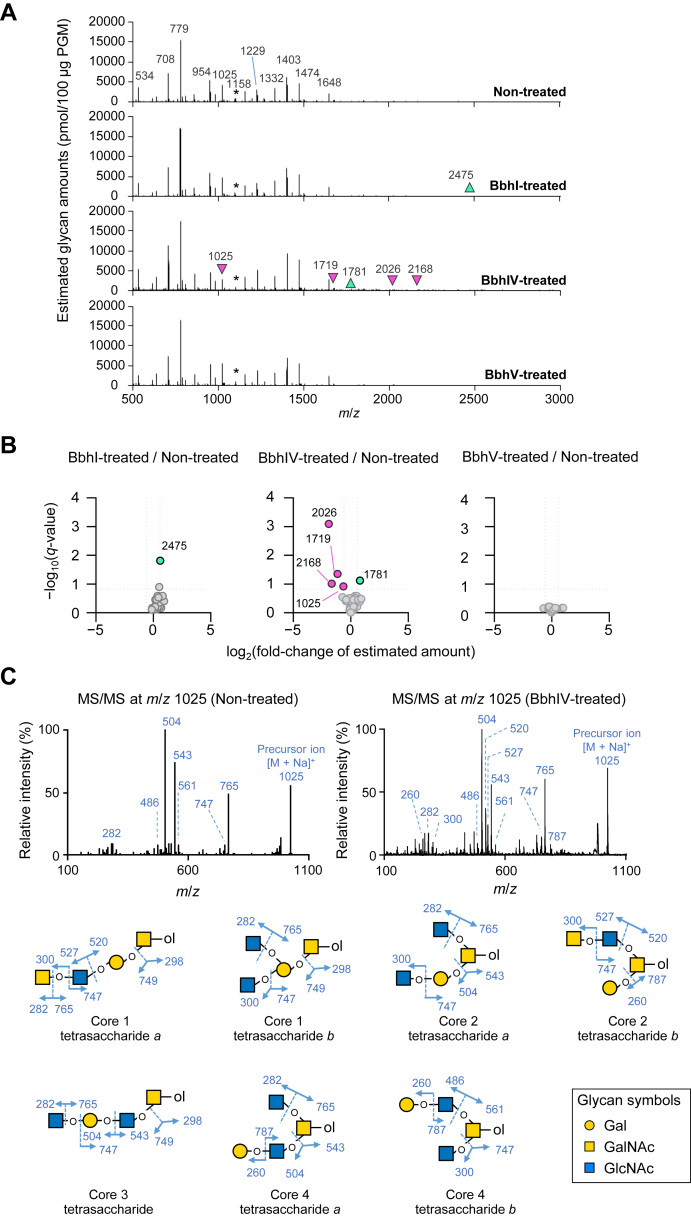
**Actions of purified BbhI, BbhIV, and BbhV on *O*-glycans of porcine gastric mucin (PGM).***A*, the representative full mass spectra of permethylated nonsulfated *O*-glycan alditols (*m*/*z* 500–3000) obtained from PGM incubated with the purified recombinant BbhI, BbhIV, or BbhV for 24 h. Nontreated PGM was also analyzed for comparison. *Pink and light green arrowheads* indicate the ion peaks whose estimated amounts decreased and increased, respectively, upon enzymatic treatment (see Table S1 for glycan composition and semiquantification). *Asterisks* indicate LNFP I added as an external control. *B*, volcano plots comparing the estimated amount of each *O*-glycan peak obtained from the BbhI, BbhIV, or BbhV-treated sample with that of the nontreated sample. Data obtained in three independent experiments were used for the analysis. Fold changes and their *q*-values were used for plotting to identify peaks whose abundances changed by more than 1.5-fold with *q*-values of < 0.15 post treatment. The *q*-values are the adjusted *p*-values obtained by multiple *t* tests with false discovery rate correction (74). The *m*/*z* values of the peaks of interest are shown. *C*, tandem mass spectra (MS/MS) of the *m*/*z* 1025 peak obtained from the nontreated (*left panel*) and BbhIV-treated (*right panel*) samples. Predicted *O*-glycan isomers are shown with their fragmentation patterns. Glycan symbols are depicted according to the nomenclature committee of the Consortium for Functional Glycomics (72).

Next, we performed semiquantitative *O*-glycomic analysis of PGM after incubation with the purified recombinant enzymes and compared the estimated amount of each *O*-glycan with that obtained from the nontreated control sample (Fig. 2*A*). We separated nonsulfated *O*-glycans from sulfated *O*-glycans and used the nonsulfated *O*-glycans for analysis. A volcano plot with false discovery rate correction showed that, among the 38 *O*-glycan peaks detected as permethylated alditols (sodium adducts) (Supporting Information Table S1), the estimated amounts of four ion peaks of *m*/*z* 1025, 1719, 2026, and 2168 decreased by more than 1.5-fold upon BbhIV treatment, with *q* values of less than 0.15 (Fig. 2*B*). Tandem mass spectrometry (MS/MS) analysis of the ion peak at *m*/*z* 1025 (Hex1HexNAc2GalNAc-itol) obtained from the nontreated sample proposed one possible structure, core 2 tetrasaccharide *a*, as a major *O*-glycan species. Fragment ion peaks diagnostic of core 1- and core 3-type tetrasaccharides, *i.e.*, *m*/*z* 298 and 749, and core 2 tetrasaccharide *b* and core 4 tetrasaccharide *a*/*b*, *i.e.*, *m*/*z* 260 and 787, were not detected (Fig. 2*C*, left panel). Upon treatment with BbhIV, the fragment ions at *m*/*z* 260 and 787 appeared (Fig. 2*C*, right panel). In addition, the fragment ions at *m*/*z* 520 and 527, which are indicative of core 2 tetrasaccharide *b*, were detected. The fragment ions at *m*/*z* 298 and 787 were not detected. These results indicate that the relative abundance of core 2 tetrasaccharide *a* decreased among the *O*-glycan species of *m*/*z* 1025. All taken together, it is highly likely that BbhIV trimmed a terminally attached β-(1→6)-GlcNAc residue of core 2 tetrasaccharide *a*, although its action on this short *O*-glycan seems to be limited due to densely packed *O*-glycans on PGM. MS/MS analysis of the ion peaks at *m*/*z* 1719, 2026, and 2168 obtained from the nontreated sample indicated the presence of a terminally attached HexNAc residue based on the formation of fragment ions at *m*/*z* 1459, 1766, and 1908, respectively (−260 Da for HexNAc) (Supporting Information Fig. S2). These fragment ions disappeared or their intensities relative to the other fragment ions decreased upon BbhIV treatment, suggesting the removal of a terminal HexNAc residue upon BbhIV treatment. The estimated amount of an ion peak of *m*/*z* 1781 (Fuc2Hex3HexNAc2GalNAc-itol) increased by more than 1.5-fold upon BbhIV treatment. Although no diagnostic fragmentation pattern was observed for this peak pre- and post-BbhIV treatment, this glycan could be formed from a glycan of *m*/*z* 2026 (Fuc2Hex3HexNAc3GalNAc-itol).

The estimated amount of one peak at *m*/*z* 2475 increased by incubating PGM with BbhI. We did not analyze this peak further because of its compositional complexity and low abundance. No remarkable alterations were detected for BbhV-treated PGM samples compared with the control. Overall, these results suggest that, although the linkages susceptible to BbhI, BbhIV, and BbhV are scarcely exposed at the surface of PGM, BbhIV hydrolyzes a terminally attached β-(1→6)-GlcNAc residue.

### Roles of BbhI, BbhIV, and BbhV in mucin *O*-glycan degradation

**Figure 3:**
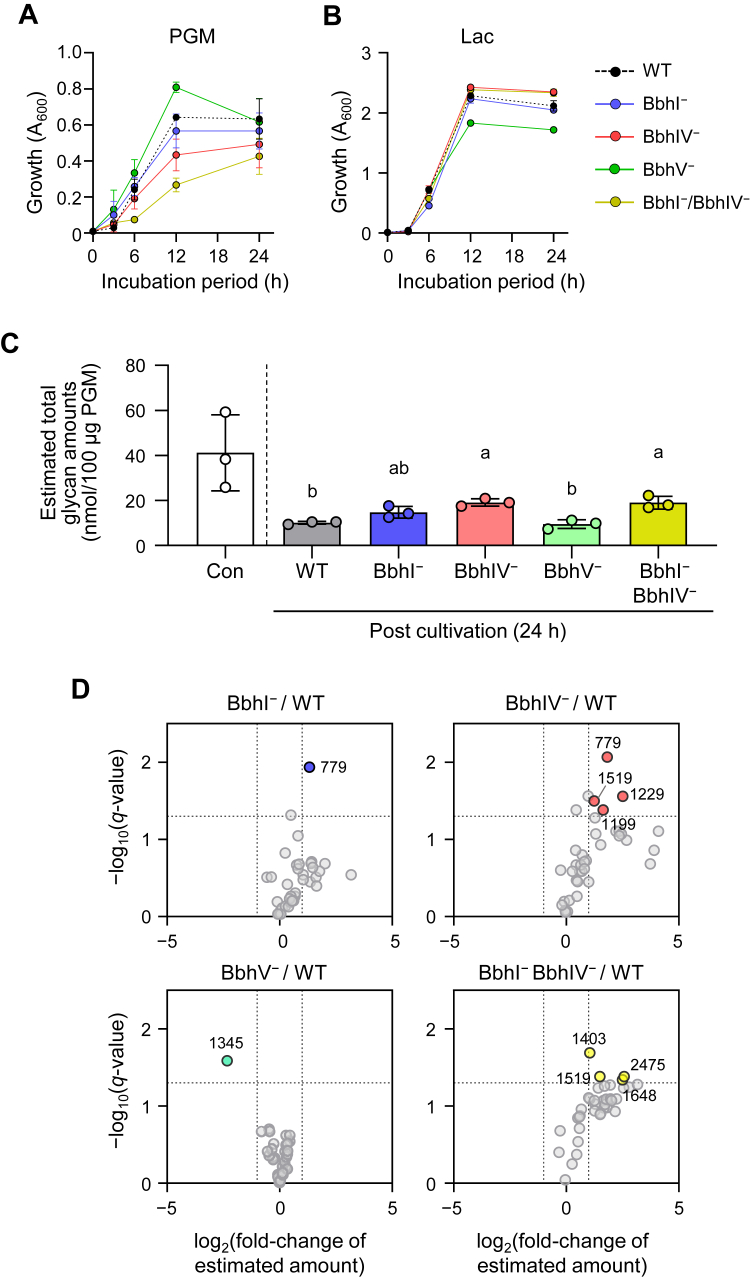
**Porcine gastric mucin (PGM) *O*-glycan degrading ability differs among the WT, BbhI**^**−**^**, BbhIV**^**−**^**, BbhV**^**−**^**, and BbhI**^**−**^**/BbhIV**^**−**^**strains of *B. bifidum*.***A* and *B*, the growth of wildtype (WT) and mutant (BbhI^−^, BbhIV^−^, BbhV^−^, and BbhI^−^/BbhIV^−^) strains of *B. bifidum* JCM 1254 in the modified de Man-Rogosa-Sharpe medium supplemented with 0.5% PGM (*A*) or Lac (*B*). The circles and whiskers represent mean ± SD of three independent experiments. Symbols used are indicated on the *right*. *C*, estimated total amounts of *O*-glycans obtained from PGM incubated with *B. bifidum* variants for 24 h (see Table S2 for glycan composition and semiquantification). The bars and whiskers represent mean ± SD of three independent experiments. One-way ANOVA followed by Tukey’s multiple comparison test was used for the statistical analysis. Different letters (a and b) indicate significant differences among the groups (*p* < 0.05). *p*-Values are shown in Fig. S5. Note that the noninoculated sample was excluded from the statistical analysis. *D*, volcano plots comparing the estimated amount of each of *O*-glycan peaks obtained from BbhI^−^, BbhIV^−^, BbhV^−^, or BbhI^−^/BbhIV^−^-inoculated PGM sample with that obtained from WT-inoculated sample. Fold changes and their *q*-values were used for plotting to identify peaks whose abundances changed by more than 2-fold with *q*-values of less than 0.05. The *q*-values are the adjusted *p*-values obtained by multiple *t* tests with false discovery rate correction (74). The *m*/*z* values of the peaks of interest are shown.

To further elucidate the functional difference between the three β-*N*-acetylglucosaminidases in mucin *O*-glycan breakdown by *B. bifidum*, we disrupted each enzyme (BbhI^−^, BbhIV^−^, and BbhV^−^) (Supporting Information Fig. S3). We also constructed a double mutant (BbhI^−^/BbhIV^−^) in anticipation of observing a stronger phenotypic change than that observable for the three single mutants, as BbhI and BbhIV were inducible by PGM and exhibited distinct linkage preferences for core linkages of *O*-glycans (Fig. 1, *A*, *C*, and *D*). First, the growth properties of these mutants were examined by comparing the area under the growth curves (AUC) (Fig. 3, *A* and *B*). Growth on Lac was comparable between wildtype (WT), BbhI^−^, and BbhIV^−^ strains (within ±1.1-fold). However, when PGM was used as a carbon source, the BbhIV^−^ mutant showed decreased growth compared with the WT strain, with its AUC being 73% of WT. The growth of the BbhI^−^ strain on PGM was comparable with that of the WT strain (93%). Compared with the WT strain, the AUCs of the BbhV^−^ strain grown on Lac and PGM were 83 and 120%, respectively. The reasons for the apparent decrease and increase in growth rates on Lac and PGM, respectively, observed for the BbhV^−^ mutant is unclear. Overall, these results demonstrated the marked contribution of BbhIV to mucin *O*-glycan assimilation by the bacterium. Interestingly, the importance of BbhI in PGM *O*-glycan degradation became apparent in the double mutant (BbhI^−^/BbhIV^−^). The AUC of the double mutant on PGM was half (51%) of that of WT, while the values were comparable between the two strains when grown in Lac-supplemented medium.

PGM *O*-glycans were then analyzed using the samples collected post 24-h cultivation. The full mass profiles showed different patterns between the noninoculated and *B. bifidum*–inoculated samples (Supporting Information Fig. S4 and Table S2). The estimated total *O*-glycan amount was 41.2 nmol per 100 μg of PGM extracted from the control culture, while it was 10.1 nmol per 100 μg of PGM extracted from the culture incubated with WT *B. bifidum* (Fig. 3*C*). The estimated total amount of PGM *O*-glycans obtained from WT-inoculated culture was comparable with that obtained from BbhV^−^-inoculated culture (9.5 nmol per 100 μg of PGM) (Tukey's multiple comparisons test) (see Supporting Information Fig. S5 for *p*-values). In contrast, higher amounts of *O*-glycans remained untrimmed in the cultures of BbhIV^−^- and BbhI^−^/BbhIV^−^-inoculated samples (19.1 and 19.0 nmol per 100 μg of PGM, respectively) than in the WT-inoculated culture. The degree of *O*-glycan degradation by the BbhI^−^ strain (14.8 nmol per 100 μg of PGM) was at an intermediate level between those observed for WT and BbhIV^−^. The varied glycan trimming capabilities of these strains were consistent with the difference observed in their growth ability at 24 h. Note that statistical analysis was performed to compare the glycan amounts of *B. bifidum*–inoculated samples, and the analysis did not include the control.

**Figure 4:**
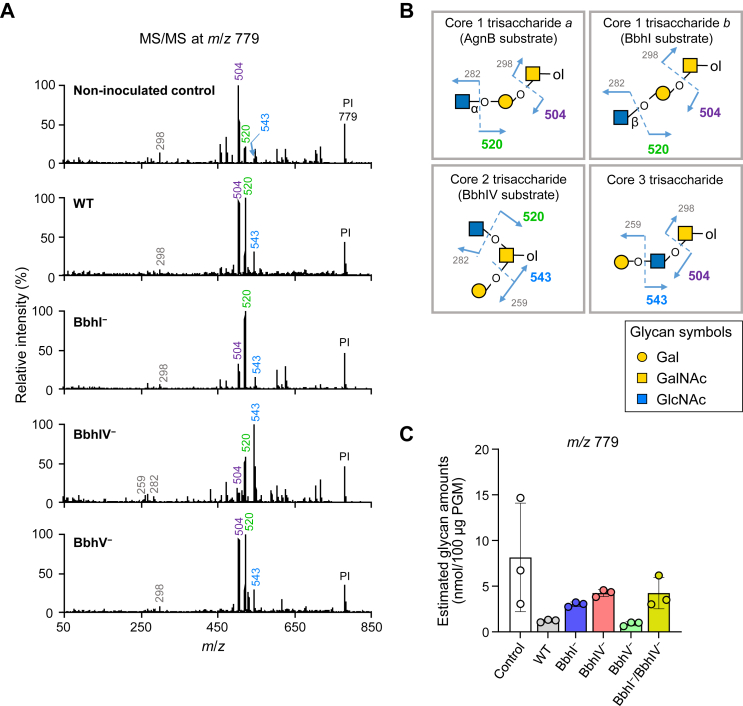
**Tandem mass spectrometry (MS/MS) analysis of the ion peaks at *m/z* 779 obtained from porcine gastric mucin (PGM) after incubation with *B. bifidum* variants.***A*, different MS/MS spectra were obtained for the *m*/*z* 779 peaks (precursor ion, PI) in *O*-glycomic analysis of PGM incubated with WT, BbhI^−^, BbhIV^−^, and BbhV^−^ strains of *B. bifidum* for 24 h (related with Fig. 3*D*). Diagnostic fragment ion peaks are shown in different colors. *B*, the four isomeric *O*-glycan structures predicted from MS/MS analysis are shown with their fragmentation patterns. *C*, estimated amounts of the *m*/*z* 779 peak obtained from noninoculated and *B. bifidum*-inoculated samples (see Table S2). Glycan symbols are depicted according to the nomenclature committee of the Consortium for Functional Glycomics (72).

Volcano plots with false discovery rate correction were used to identify PGM *O*-glycan species whose abundance significantly changed (fold change of > 2 and *q*-values of < 0.05) upon the loss of each of the three β-*N*-acetylglucosaminidases (Fig. 3*D*). Estimated amounts of one ion peak at *m*/*z* 779; four ion peaks at *m*/*z* 779, 1199, 1229, and 1519; and four ion peaks at *m*/*z* 1403, 1519, 1648, and 2475 increased after incubation with BbhI^−^, BbhIV^−^, and BbhI^−^/BbhIV^−^ strains as compared with WT-inoculated sample, respectively, while an ion peak at *m/z* 1345 decreased after incubation with the BbhV^−^ strain compared with WT-inoculated sample. MS/MS analysis of the peak at *m/z* 779 (Hex1HexNAc1GalNAc-itol) derived from the noninoculated control sample (Fig. 4*A*) proposed four isomeric glycan species, *i.e.*, the core 1 structure with a terminal α- or β-linked GlcNAc (core 1 trisaccharide *a*/*b*), a core 2 trisaccharide, and a core 3 trisaccharide with a terminal Gal (Fig. 4*B*). The presence of α-GlcNAc or β-GlcNAc residue at the nonreducing ends of PGM *O*-glycans has been reported previously (32, 42). Interestingly, MS/MS analysis gave different fragmentation patterns among *B. bifidum*–inoculated samples (Fig. 4*A*). In the noninoculated sample, the two most abundant fragment peaks were at *m*/*z* 504 and 520, followed by the peak at *m*/*z* 543, suggesting that the glycan peak comprises the core 1 structures as a major species. However, after cultivation with the BbhIV^−^ strain, the fragment ion at *m*/*z* 543 became predominant, followed by the peak at *m*/*z* 520. The *m*/*z* 543 fragment ion can be produced from core 2 and core 3 trisaccharides but not from core 1 structures, and the *m*/*z* 520 peak is not formed from core 3 trisaccharide. Therefore, the peak of *m/z* 779 that was left unconsumed in the BbhIV^−^-inoculated sample should represent a core 2 trisaccharide. In the BbhI^−^-culture, a fragment ion at *m*/*z* 520, which can be produced from core 1 and core 2 trisaccharides, became predominant, followed by the peaks at *m/z* 504 and *m/z* 543. The results suggested that either or both of the core 1 trisaccharides *a* and *b* remained mostly untrimmed in PGM *O*-glycans in the BbhI^−^-inoculated culture. Supposing that the α-linked GlcNAc in core 1 trisaccharide *a* is removed by an α-*N*-acetylglucosaminidase AgnB (32), the remaining species could be core 1 trisaccharide *b*. The fragmentation pattern of the peak at *m/z* 779 obtained from the BbhV^−^-inoculated sample was the same as that obtained from the WT-inoculated sample, indicating a trivial function of BbhV in the *O*-glycan trimming by *B. bifidum*. The peak of *m/z* 779 also accumulated in the BbhI^−^/BbhIV^−^ strain at a comparable level with the BbhIV^−^ strain (Fig. 4*C*). Loss of BbhI activity might be partly complemented by BbhV, as BbhV was able to hydrolyze the GlcNAcβ1-3Gal linkage that is present in mucin *O*-glycans as an *N*-acetyllactosamine (LacNAc) unit (Fig. 1*H*) (43).

MS/MS analysis of the peak at *m/z* 1229, whose estimated abundance increased in the culture with the BbhIV^−^ strain (Fig. 3*D*), also indicates the involvement of BbhIV in the removal of the β-(1→6)-linked GlcNAc residue, as the MS/MS spectra obtained from the BbhIV^−^-inoculated sample did not contain a fragment ion of *m/z* 953 that is formed only from core 1 and core 3 pentasaccharides but instead contained fragment ions of *m*/*z* 543 and 708 that can be formed from a core 2 pentasaccharide *b* (Supporting Information Fig. S6). A core 4 pentasaccharide does not produce *m*/*z* 543 and 708 fragment ions, either. Thus, the ion peak at *m*/*z* 1229 could contain the core 2 pentasaccharide *b* structure that remained untrimmed due to the lack of BbhIV. The other ion peaks whose abundances significantly changed upon incubation with single and double mutants as compared with the WT strain (Fig. 3*D*) were not analyzed further because of the complexities or low quantities of the glycans. The *O*-glycans left unconsumed, with statistical significance, seemed to have higher *m*/*z* values (longer chains) in the double mutant–inoculated samples than in the other single mutant–inoculated samples (Fig. 3*D*).

### Efficient degradation of the mucin core 4 structure requires an ordered action of BbhIV and BbhI

**Figure 5:**
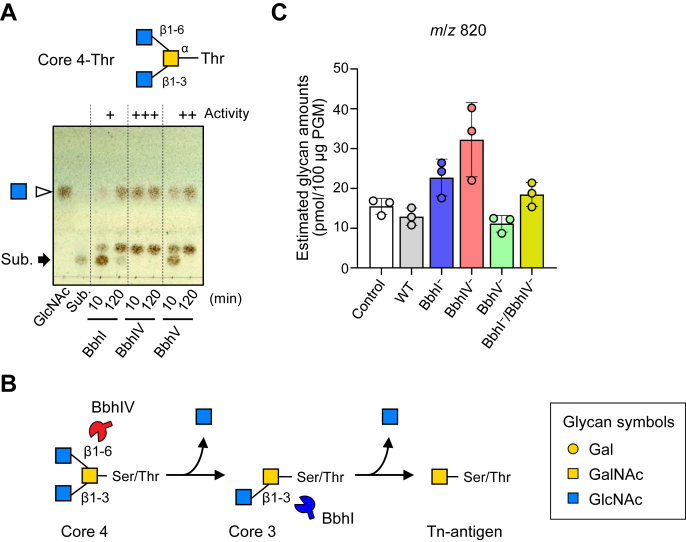
**Degradation of mucin core 4 structure requires an ordered action of BbhIV and BbhI.***A*, TLC analysis of the reaction products. Mucin core 4-Thr (4 mM) was incubated with the purified recombinant enzyme (1 μM of BbhI, BbhIV, or BbhV) for 10 min and 120 min. An arrow indicates the substrate. GlcNAc was used as a standard. See Table 1 footnote for activity evaluation expressed by +/−. *B*, a proposed degradation scheme of the mucin core 4 structure by *B. bifidum*. Prior removal of the β-(1→6)-GlcNAc residue by BbhIV is required for the efficient hydrolysis of the β-(1→3)-GlcNAc linkage by BbhI. The resulting Tn-antigen is assumed to be internalized into the cells by an unidentified transporter (20). *C*, estimated amounts of the *m/z* 820 peak (HexNAc2GalNAc-itol) obtained from porcine gastric mucin (PGM) with and without incubation with *B. bifidum* variants. Glycan symbols are depicted according to the nomenclature committee of the Consortium for Functional Glycomics (72).

Given the physiological importance of BbhI and BbhIV in mucin *O*-glycan breakdown (Figs. 3 and 4), we then examined how *B. bifidum* utilizes these β-*N*-acetylglucosaminidases with distinct linkage preferences to degrade *O*-glycan structures that possess both β-(1→3)- and β-(1→6)-GlcNAc residues attached with the same proximal residue. To this end, we incubated the core 4-Thr with the purified enzymes (Fig. 5*A*). TLC analysis showed that the rate of hydrolysis of the β-(1→3)-linkage in the core 4 structure by BbhI is considerably slower than that of the same linkage in the core 3 structure (Fig. 1*C* *versus* Fig. 5*A*). On the other hand, BbhIV with a β-(1→6)-linkage preference efficiently released GlcNAc from the core 4-Thr structure. The activity of BbhV for the β-(1→6)-GlcNAc linkage in the core 4-Thr structure was weaker than that of BbhIV. Thus, degradation of *O*-glycans with two branched GlcNAc residues requires the coordinated action of BbhI and BbhIV, and prior removal of the β-(1→6)-GlcNAc residue by BbhIV is pivotal for subsequent hydrolysis of the β-(1→3)-GlcNAc linkage by BbhI to occur (Fig. 5*B*). The estimated glycan amount of the ion peak of *m*/*z* 820 (HexNAc2-GalNAc-itol) increased after incubation with the BbhIV^–^ strain, although statistically insignificant (Fig. 5*C*). We did not conduct the fragmentation analysis of this peak due to low abundance, but it is possible that the core 4 trisaccharide structure accumulated on PGM after incubation with the mutant. The peak detected from the noninoculated sample might represent a core 3 trisaccharide structure with an *N*,*N′*-diacetyllactosamine unit (GalNAcβ1-4GlcNAcβ1-3GalNAcα-*O*-Ser/Thr) (42), which is resistant to all secretory GHs of *B. bifidum* as the glycan amount detected was similar between the control and WT-inoculated samples. In the BbhIV^–^ strain, other secretory GHs could trim some *O*-glycans to expose the core 4 structure, which might cause accumulation of the ion peak of *m*/*z* 820. Neither the BbhI^−^ nor the BbhI^−^/BbhIV^−^ mutant might be able to trim PGM *O*-glycans to expose the core 4 trisaccharide structure, which could result in lower accumulation of the glycan than that detected for the BbhIV^–^ mutant.

### Loss of BbhI and BbhIV affects the cross-feeding ability of *B. bifidum*

**Figure 6:**
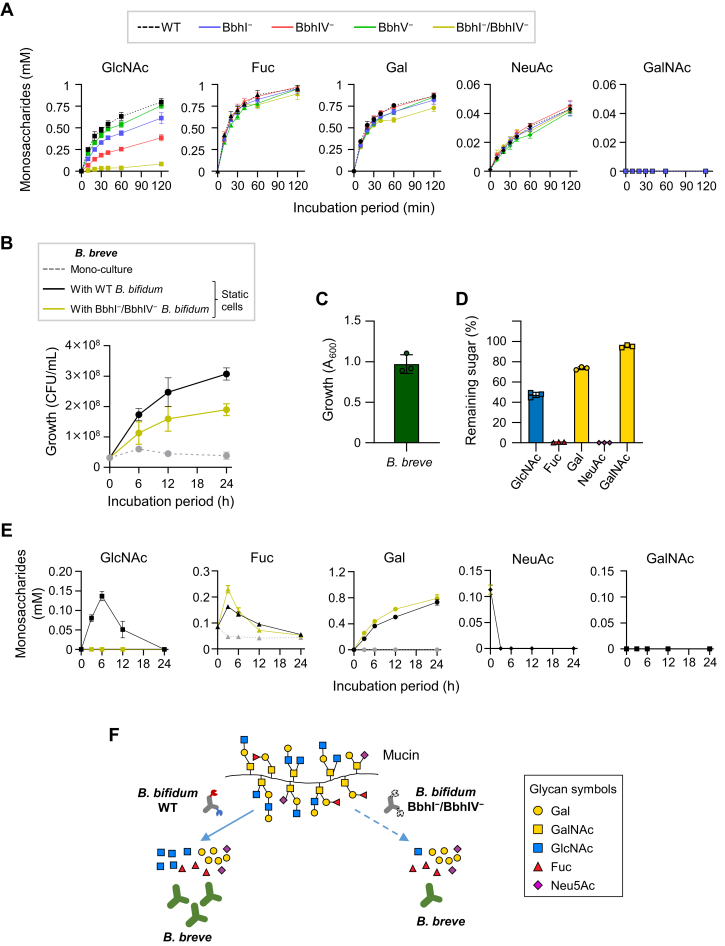
**Lack of BbhI and BbhIV affects cross-feeding behavior of *B. bifidum* primarily by affecting the ability to release GlcNAc from mucin *O*-glycans.***A*, the rate of monosaccharide release into the culture supernatant by the WT, BbhI^−^, BbhIV^−^, BbhV^−^, and BbhI^−^/BbhIV^−^*B. bifidum* strains. The chemically de-energized cells were incubated in the modified de Man-Rogosa-Sharpe medium supplemented with 0.5% porcine gastric mucin. Monosaccharides were quantified as described under the Experimental procedures section. Symbols and whiskers represent mean ± SD of three independent experiments, with different colors used for respective strains shown above the *panel*. *B*, the growth (CFUs) of *B. breve* in the porcine gastric mucin–supplemented medium. *B. breve* was incubated without (*dashed gray line*) or with *B. bifidum* WT (*black line*) or BbhI^−^/BbhIV^−^ strain (*yellow line*). Tetracycline was added to the medium to make *B. bifidum* cells static. Tet^R^ was conferred to *B. breve* by introducing pMSK219. Circles and whiskers represent the mean ± SD of three independent experiments. (C and D) The growth of *B. breve* on *O*-glycan-constituent monosaccharides (GlcNAc, Fuc, Gal, NeuAc, and GalNAc: 0.2% each). *C*, the *A*_600_ value obtained post 24-h cultivation. The initial *A*_600_ value was 0.01. *D*, sugar consumption by *B. breve* post 24-h cultivation. Sugars were analyzed by high-performance anion exchange chromatography with pulsed amperometric detection. Bar and whiskers represent mean ± SD of three independent experiments. *E*, *O*-glycan-constituent monosaccharide concentrations in the culture supernatant of the *B. breve* monocultivated medium and cocultivated medium with *B. bifidum* WT or BbhI^−^/BbhIV^−^ strain. Symbols and whiskers represent mean ± SD of three independent experiments. Colors used are as in (*B*). *F*, lowered GlcNAc-releasing ability due to the lack of BbhI and BbhIV affects the cross-feeding behavior of *B. bifidum*.

*B. bifidum* degrades host-derived glycans extracellularly and cross-feeds the degradant products to other gut microbes (44). We examined the effect that the disruption of the *bbhI*, *bbhIV*, and/or *bbhV* gene(s) had on the ability of *B. bifidum* to liberate monosaccharides from mucin *O*-glycans. The disaccharide lacto-*N*-biose I (Galβ1-3GlcNAc), which is released by the action of LnbB, was not quantified due to its instability (45). The cell suspensions of WT and mutant strains were incubated in the presence of PGM, and monosaccharides released in the supernatant were quantified (Fig. 6*A*). To avoid assimilation of the released sugars by *B. bifidum*, the cells were de-energized by a proton uncoupler and an ABC transporter inhibitor prior to incubation (46). While neither Fuc nor NeuAc liberation was affected by any of β-*N*-acetylglucosaminidase inactivation, Gal liberation seemed to be slightly reduced for the BbhI^−^/BbhIV^−^ double mutant. As expected, the ability to release GlcNAc differed markedly among the mutants. Inactivation of BbhIV alone decreased the ability of the cells to release GlcNAc to half of that of the WT strain (0.39 mM *versus* 0.80 mM at 120 min). Decreased GlcNAc liberation was also observed for the BbhI^–^ mutant (0.61 mM at 120 min), while BbhV inactivation did not have a notable effect. The most pronounced effect was observed for the BbhI^–^/BbhIV^–^ double mutant. GlcNAc liberation activity was 10-fold less than that observed for the WT strain (0.085 mM *versus* 0.80 mM at 120 min). These results are consistent with the growth behaviors observed for the mutants on PGM and may also reflect the data obtained in the *O*-glycomic analyses, in which more *O*-glycans remained unconsumed when incubated with BbhIV^−^ and BbhI^−^/BbhIV^−^ mutants than when incubated with WT, BbhI^−^, or BbhV^−^ mutants (Fig. 3*C*).

We then examined how the diminished ability of the double mutant to release GlcNAc from mucin *O*-glycans affects the cross-feeding ability of *B. bifidum*. *Bifidobacterium breve* JCM 1192^T^ was used for cocultivation, as this strain is incapable of growing on PGM (Fig. 6*B*). *B. breve* grew well in a medium supplemented with a mixture of monosaccharides (0.2% each) that constitute PGM *O*-glycans (Fig. 6*C*), in which the species consumed Fuc and NeuAc completely, GlcNAc moderately (50%), and Gal slightly (25%) (Fig. 6*D*). GalNAc was not utilized by the strain. Upon cocultivation with the WT strain of *B. bifidum* in a PGM-supplemented medium, *B. breve* was able to proliferate, and time-dependent consumption of Fuc, NeuAc, and GlcNAc was observed (Fig. 6*E*). Note that tetracycline (Tet) was added to the medium to make *B. bifidum* cells static during cocultivation (47). *B. breve* was transformed with a plasmid carrying the Tet resistance gene (see the Experimental procedures section). The inability of *B. bifidum* to utilize Fuc and NeuAc has been previously shown (19, 33, 40, 48); therefore, the growth of *B. breve* is attributable to *B. bifidum*–mediated cross-feeding of these monosaccharides. Most of the released GlcNAc could be utilized by *B. breve*, although static *B. bifidum* cells might have metabolized a small amount of GlcNAc. Gal continuously accumulated during cocultivation (Fig. 6*E*). Neither *B. breve* nor *B. bifidum* encodes a Gal transporter gene in their genomes (40, 49). GalNAc was not detected throughout the incubation period, as no extracellular *N*-acetylgalactosaminidase activity has been reported for *B. bifidum*. When coincubated with the *B. bifidum* BbhI^−^/BbhIV^−^ strain, the colony-forming units of *B. breve* decreased by about half (61% of WT in AUC) (Fig. 6*B*). As a reflection of the low ability of the double mutant to release GlcNAc from PGM *O*-glycans, the GlcNAc concentration in the cocultivation medium was negligible during the incubation period. The concentrations of other sugars in the medium were similar to those observed when coincubated with WT *B. bifidum*, although the Fuc concentration seemed to be temporarily higher during the early growth, which might be due to a slow growth rate of *B. breve* coincubated with the mutant *B. bifidum*. Provided that the biomass of *B. bifidum* cells that were made static by adding Tet did not differ between WT and BbhI^–^/BbhIV^–^ strains during cocultivation, the lowered growth of *B. breve* can be considered to be primarily caused by the lowered ability of the *B. bifidum* mutant to release GlcNAc from PGM *O*-glycans (Fig. 6*F*).

### Phylogenetic analysis of GH84 enzymes

**Figure 7:**
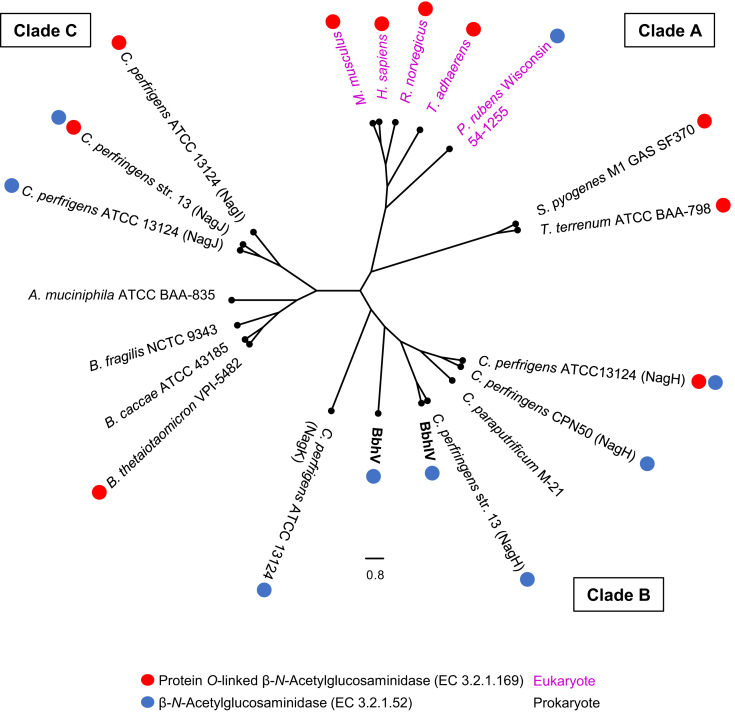
**Phylogenetic analysis of GH84 catalytic domains.** The tree was created using GH84 catalytic domains of characterized enzymes (38), BbhIV and BbhV of *B. bifidum*, and uncharacterized enzymes of several mucin-degrading gut microbes. UniProt IDs of the homologs used for tree construction are AAH39583.2 (*Homo sapience*); AAG43273.2 (*Mus musculus*); CAP80500.1 (*Penicillium rubens* Wisconsin 54-1255); AAK72103.1 (*Rattus norvegicus*); AKA09674.1 (*Trichoplax adhaerens*); AAO79500.1 (*Bacteroides thetaiotaomicron* VPI-5482); BAC99989.1 (*Clostridium paraputrificum* M-21); ABG83307.1, ABG84510.1, ABG84519.1, and ABG84775.1(*Clostridium perfringens* ATCC 13124); AAA23259.1 (*Clostridium perfringens* CPN50); BAB79897.1 and BAB80940.1 (*Clostridium perfringens* str. 13); AAK34378.1 (*Streptococcus pyogenes* M1 GAS SF370); ACZ41038.1 (*Thermobaculum terrenum* ATCC BAA-798); ACD03898.1 (*Akkermansia muciniphila* ATCC BAA-835); ASM65960.1 (*Bacteroides caccae* ATCC 43185); CAH09476.1 (*Bacteroides fragilis* NCTC 9343); GJM46876.1 (BbhIV); and GJM47429.1 (BbhV). The catalytic domains were identified by the Protein Families Database (70). The sequences were aligned using Clustal Omega (71), and the tree was constructed by FigTree v1.4.4. The reported activities are shown by red and blue circles for protein *O*-linked β-*N*-acetylglucosaminidase and β-*N*-acetylglucosaminidase, respectively (38). Eukaryotic and bacterial members are shown by purple and black letters, respectively.

A phylogenetic tree was created using GH84 catalytic domains of the characterized enzymes (34, 38). BbhIV and BbhV from *B. bifidum* and other homologs from selected mucinolytic gut microbes (*Akkermansia muciniphila* and several *Bacteroides* species) were also included for the tree construction (Fig. 7). The analysis revealed high sequence diversity among the homologs that resulted in the formation of three distinct clades termed A, B, and C. Interestingly, clade A, which mainly consists of eukaryotic enzymes and thus represents protein *O*-linked β-*N*-acetylglucosaminidases, contains bacterial homologs from *Streptococcus pyogenes* and *Thermobaculum terrenum*, for which corresponding activity has been reported (50, 51). The four paralogs of *C. perfringens* with 23 − 64% identity were classified into two different clades, either with BbhVI and BbhV (clade B) or with *A. muciniphila* and *Bacteroides* species homologs (clade C). Clade B consists of enzymes from *B. bifidum* and *C. perfringens* and could represent β-*N*-acetylglucosaminidases that are primarily involved in host glycan degradation, although protein *O*-linked β-*N*-acetylglucosaminidase activity was reported for NagH from *C. perfringens* ATCC13124 (34). The ability of *C. perfringens* to grow on mucin has been reported *in vitro* (52). Clade C comprises homologs from *Bacteroides* spp., *A. muciniphila*, and *C. perfringens*, and both β-*N*-acetylglucosaminidase and protein *O*-linked β-*N*-acetylglucosaminidase activities have been reported for these homologs. The tree suggests that GH84 members have diversified to acquire different activities, possibly through horizontal gene transfer events not only between microbes but also between host and microbes.

## Discussion

Although many *in vitro* studies have shown the ability of *B. bifidum* to degrade mucin *O*-glycans, its *in vivo* mucinolytic activity had been controversial (53). We have recently shown that *B. bifidum* does degrade intestinal mucin *O*-glycans using a gnotobiotic mouse model, in which the amounts of fecal *O*-glycans decreased while the fecal concentrations of mucin *O*-glycan constituent monosaccharides increased upon administration of *B. bifidum*. The study also compared fecal *O*-glycan structures obtained from WT-monocolonized mice with those obtained from BbhII^‒^-monocolonized mice, revealing that a sulfoglycosidase BbhII plays a pivotal role in degrading intestinal sulfated *O*-glycans (19). In the present study, we focused on three extracellular *N*-acetylglucosaminidases of *B. bifidum* and showed that BbhI (GH20) and BbhIV (GH84) with different linkage preferences play important roles in degrading mucin *O*-glycans. BbhI hydrolyzes the β-(1→3)-GlcNAc linkage of the core 3 structure that is widely present in the gastrointestinal tracts (3). The enzyme is probably also involved in hydrolyzing the β-(1→3)-GlcNAc linkage in the LacNAc unit of elongated sugar chains. In contrast, BbhIV is responsible for hydrolyzing the β-(1→6)-GlcNAc linkages of mucin core 2 and core 4 structures that are found in the distal colon and ileum, respectively. It presumably hydrolyzes the β-(1→6)-GlcNAc linkages present in the branching points inside the sugar chains (3). Our results also revealed that a sequential action by BbhIV followed by BbhI is required for efficiently degrading branched *O*-glycans with both β-(1→3)- and β-(1→6)-GlcNAc residues (Fig. 5). The total amounts of the remaining *O*-glycans were highest in BbhIV^−^-inoculated samples, which suggests that, similarly to BbhI, other enzymes such as β-galactosidases are not able to trim *O*-glycans when they encounter branching β-(1→6)-GlcNAc residues.

Turroni *et al*. previously performed a transcriptome analysis of *B. bifidum* PRL2010 and reported that various GH genes including BBPR_1529 (a BbhI homolog), BBPR_1018 (a BbhII homolog), and BBPR_1514 (a BbhIV homolog) were upregulated when grown on PGM (54). Our analysis also showed the upregulation of *bbhI*, *bbhII*, and *bbhIV* by PGM. In addition to PGM, *B. bifidum* is known to utilize HMOs, which share structural similarity to mucin *O*-glycans (44, 55). Recently, Arzamasov *et al.* revealed that NagR, a ROK family transcriptional regulator, governs the expression of genes involved in the utilization of GlcNAc-containing HMOs in *B. longum* subsp. *infantis* and proposed a NagR-binding motif sequence (56). Interestingly, the NagR binding motif-like sequences are present in the upstream regions of *bbhII* and *bbhIV* of *B. bifidum* but are absent in the upstream regions of the *bbhI* and *bbhV* genes. The fold change in upregulation was similar between *bbhII* and *bbhIV*, while the *bbhI* induction level was considerably high compared with that observed for *bbhII* and *bbhIV*. The extent of upregulation for *bbhV* was very low (Fig. 1*A*). Thus, while *bbhII* and *bbhIV* could be members of the NagR regulon, *bbhI* was under the control of a different regulator in *B. bifidum*. NagR regulon could be a part of the complex host glycan utilization system working in *B. bifidum*.

Disruption of the *bbhI* and *bbhIV* genes did not affect the Fuc and NeuAc liberation ability of *B. bifidum* (Fig. 6*A*). This is because Fuc and NeuAc modifications essentially occur at the nonreducing termini of *O*-glycans. On the other hand, the Gal trimming ability was slightly reduced in the double mutant. The lack of BbhI and BbhIV might slightly affect the efficient degradation of the LacNAc unit in PGM *O*-glycan structures (42). As mentioned above, BbhV might partially compensate for the lack of BbhI. A decrease in the ability to release GlcNAc was observed for BbhI^−^, BbhIV^−^, and BbhI^−^/BbhIV^−^ strains to different extents (Fig. 6*A*). The double mutation not only impaired the growth ability of *B. bifidum* on PGM (Fig. 3*A*) but also decreased the cross-feeding ability to other microbes, as shown in the cocultivation experiments using *B. breve* (Fig. 6*B*).

Since the discovery of protein *O*-linked β-*N*-acetylglucosaminylation (*O*-GlcNAcylation) in higher eukaryotes and the following identification of a human GH84 homolog that catalyzes de-GlcNAcylation of modified proteins (36), GH84 members have attracted significant attention as a key enzyme that modulates *O*-GlcNAcylation/phosphorylation of proteins in various cellular processes (37). Research has accelerated after reports of several bacterial GH84 members that act on *O*-GlcNAc residues attached to intact proteins extracted from mammalian cell lines (57). Oral administration of a bead-coated bacterial GH84 enzyme ameliorated chemically induced gut inflammation in mice (58). GH84 members may thus mediate the cross talk between gut microbes and the host through the regulation of protein *O*-GlcNAcylation. On the other hand, our study strongly suggests that bacterial GH84 includes a member whose function is dedicated to mucin *O*-glycan degradation and thereby contributes to gut microbial community formation, as GlcNAc serves as a good carbon and nitrogen source for most gut microbes. Although some secretory bacterial GH84 enzymes potentially affect *O*-GlcNAcylation in host epithelial cells, BbhIV has likely been evolved in *B. bifidum* for *O*-glycan breakdown.

The GH84 domains of BbhIV and BbhV share 52% identity. Although they have similar linkage preferences, higher activity on core 2 and core 4 motifs was detected for BbhIV than for BbhV. A notable difference was observed when we compared their activity using a core 2 trisaccharide carrying either Thr or *p*NP at the reducing end (Fig. 1, *D* and *E*). These results, together with the gene transcription analysis, clearly show that BbhIV is primarily relevant to mucin *O*-glycan degradation by the bacterium. BbhV might be involved in the degradation of other glycans such as *N*-glycan, as the enzyme easily hydrolyzed the disaccharide GlcNAcβ1-2Man. In other cases, the enzyme may be involved in the degradation of HMOs with β-(1→6)-GlcNAc residues. As mentioned above, a GH20 enzyme of *B. longum* subsp. *infantis* (Blon_0732) is capable of hydrolyzing β-(1→6)-linkage in HMOs, whereas BbhI (GH20) of *B. bifidum* is incapable of acting on β-(1→6)-GlcNAc residues of HMOs (29). Further enzymatic analysis using agalacto bisecting *N*-glycans and branched HMOs as substrates, combined with structure-based mutational study, is necessary to understand the role of BbhV and its functional difference from BbhIV.

Our study contributes to understanding how gut microbes specifically degrade mucin *O*-glycans and how the degradation affects the growth of other microbes. Our results also indicate the diverse functions of GH84 enzymes in higher eukaryotes, gut microbes, and host–gut microbe interactions, which warrants further enzymatic, genetic, and phenotypic studies from a bacterial viewpoint.

## Experimental procedures

### Reagents

Lacto-*N*-fucopentaose I (LNFP I: Fucα1-2Galβ1-3GlcNAcβ1-3Galβ1-4Glc), *p*-nitrophenyl 6-*O*-sulfo-β-GlcNAc (GlcNAc6Sβ-*p*NP), and GlcNAcβ-*O*-Ser were purchased from Carbosynth. GlcNAcβ-*p*NP was obtained from Nacalai Tesque, and core 2-*p*NP [Galβ1-3(GlcNAcβ1-6)GalNAcα-*p*NP] was obtained from Toronto Research Chemicals. *N*,*N*′-Diacetylchitobiose (GlcNAcβ1-4GlcNAc), core 2-Thr [Galβ1-3(GlcNAcβ1-6)GalNAcα-*O*-Thr], core 3-Thr [GlcNAcβ1-3GalNAcα-*O*-Thr], and core 4-Thr [GlcNAcβ1-3(GlcNAcβ1-6)GalNAcα-*O*-Thr] were purchased from Tokyo Chemical Industry. GalNAcβ-*p*NP, GlcNAcβ1-2Man, and porcine gastric mucin (PGM, type III) were from Sigma-Aldrich. PGM was dialyzed against water and lyophilized prior to use. Lacto-*N*-triose II (GlcNAcβ1-3Galβ1-4Glc) was prepared by digesting lacto-*N*-neotetraose (Galβ1-4GlcNAcβ1-3Galβ1-4Glc, LNnT) by *Aspergillus oryzae* β-galactosidase (Sigma-Aldrich) followed by separation with Toyopearl HW-40C gel chromatography (20 mm × 600 mm) (Tosoh). LNnT was a gift from Glycom A/S. The purity was confirmed by high-performance anion exchange chromatography (HPAEC) with pulsed amperometric detection (PAD) as described later.

### Bacterial strains and culture conditions

*B. bifidum* JCM 1254 and *B. breve* JCM 1192 were obtained from the Japan Collection of Microorganisms (RIKEN Bioresource Center). Bifidobacteria were routinely cultivated in Gifu Anaerobic Medium (GAM) (Nissui Pharmaceutical) at 37 °C under anoxic conditions using an AnaeroPack (Mitsubishi Gas Chemical) or in an anaerobic chamber, InvivO_2_ 400 (Ruskinn Technology), filled with 10% CO_2_, 5% H_2_, and 85% N_2_. mMRS medium supplemented with 0.34% (*w*/*v*) sodium ascorbate and 0.02% (*w*/*v*) cysteine-HCl was used for examining mucin *O*-glycan degradation ability of *B. bifidum*. PGM was added to mMRS medium at 0.5% (*w*/*v*). A mixture of mucin *O*-glycan-constituent monosaccharides [Fuc, Gal, GalNAc, GlcNAc, and NeuAc, each 0.2% (*w*/*v*)] was used as a carbon source for examining the sugar assimilation ability of *B. breve*. *Escherichia coli* DH5α (Toyobo) was used as a host for genetic manipulation and was grown in Luria-Bertani (LB) broth (BD) at 37 °C. When necessary, antibiotics were added to the culture broth at the following concentrations: chloramphenicol (Cm, 2.5 μg/ml for bifidobacteria and 5 μg/ml for *E. coli*), spectinomycin (Sp, 30 μg/ml for bifidobacteria and 60 μg/ml for *E. coli*), tetracycline (Tet, 5 μg/ml for bifidobacteria and *E. coli*), and ampicillin (Amp, 100 μg/ml for *E. coli*). The bacterial growth was monitored by measuring absorbance at 600 nm (*A*_600_) or determining colony-forming units (CFUs) on GAM agar plates. AUC was calculated using Microsoft Excel (Microsoft). All growth assays were carried out in biological triplicates, and mean ± standard deviation (SD) is presented unless otherwise indicated.

### Reverse transcription–quantitative polymerase chain reaction

Overnight culture of *B. bifidum* JCM 1254 was transferred to fresh mMRS medium containing 0.5% PGM or Lac at an *A*_600_ value of 0.01. The culture was incubated at 37 °C under anoxic conditions, and the cells were harvested by centrifugation at the logarithmic (*A*_600_ value of 0.4–0.6) and stationary (*A*_600_ value of 1.0–1.3) phases. The pellets were quickly washed with phosphate-buffered saline (PBS), suspended in 200 μl of RNA*later* (Thermo Fisher Scientific), and stored at 4 °C until use. Total RNAs were extracted from the cells using the NucleoSpin RNA kit (TaKaRa Bio) according to the manufacturer’s instructions, except that the cells were disrupted by vigorous shaking at 1500 rpm for 10 min in a tube containing a stainless steel bead (φ = 5 mm), a scoop (∼200 mg) of zirconium beads (φ = 0.1 mm), and RA1 buffer supplied with the kit. Shake Master Neo (Bio Medical Science) was used for disruption. After additional DNase I treatment and column purification, equal amounts of total RNAs (0.5 μg) were used for the reverse transcription with PrimeScript II 1st strand cDNA synthesis kit (TaKaRa Bio). Quantitative PCR was performed with the TB Green system (TaKaRa Bio) with thermal steps consisting of 95 °C for 30 s and 40 cycles of 95 °C for 5 s and 60 °C for 30 s. The *uvrD* gene was used as a reference (54). All of the primers used in this study are listed in Supporting Information Table S3. Specific amplification of the target genes was checked by obtaining melting curves of the amplicons.

### Recombinant protein expression and purification

BbhIV was expressed as a C-terminal hexahistidine (His_6_)-tagged protein in *E. coli*. The In-Fusion Snap Assembly Master Mix (TaKaRa Bio) was used for ligation reactions unless otherwise stated. A DNA fragment encoding 29 to 1934 amino acid residues of BbhIV was PCR-amplified using KOD-One (Toyobo) with the genomic DNA of *B. bifidum* JCM 1254 as a template. The amplified fragment was ligated with a DNA fragment amplified from pET3a (Merck-Millipore), which generated an expression plasmid pET3a-BbhIV-His_6_. After sequence confirmation, the resulting plasmid was used to transform *E. coli* BL21 (DE3) Δ*lacZ*-CodonPlus (29). Concerning BbhV, the region consisting of 35 to 1111 amino acid residues was used for expression, and pET23b (Merck-Millipore) was used as a vector. The transformants were grown in LB medium supplemented with Amp and Cm at 25 °C, and when the *A*_600_ value reached 0.4 to 0.5, isopropyl-β-d-thiogalactopyranoside was added at a final concentration of 0.1 mM. After an additional 24 h of incubation, the cells were pelleted by centrifugation and resuspended in a lysis buffer consisting of 50 mM 4-(2-hydroxyethyl)-1-piperazineethanesulfonic acid (pH 8.0), 300 mM NaCl, and 10 mM imidazole. The cells were disrupted by sonication using a Q125 sonicator (QSonica), and after the removal of cell debris by centrifugation, the cell-free extract was applied onto a Ni-nitrilotriacetic acid spin column (Qiagen). The protein was purified according to the manufacturer’s instructions and desalted using Amicon Ultracel-10 K centrifugal filters (Millipore). The recombinant proteins were further purified by anion-exchange column chromatography (Mono Q 5/50 GL), in which the protein was eluted by a linear gradient of 0 to 0.5 M NaCl in 20 mM tris(hydroxymethyl)aminomethane hydrochloride (Tris-HCl) buffer (pH 8.0). An ÄKTA pure 25 system was used. The protein purity was examined by sodium dodecyl sulfate–polyacrylamide gel electrophoresis (SDS-PAGE) followed by Coomassie Brilliant Blue R-250 staining (Quick-CBB, Wako Pure Chemical). The fractions of high purity were combined, and the protein was concentrated using Amicon Ultracel-10 K centrifugal filters. The protein concentrations were determined by measuring the absorbance at 280 nm with theoretical molar absorption coefficients of 250,170 M^−1^ cm^−1^ for BbhIV-His_6_ and 204,440 M^−1^ cm^−1^ for BbhV-His_6_. NanoPhotometer (IMPLEN) was used for the measurement. Expression and purification of BbhI (33–1604 amino acid residues) were carried out as described (29).

For native molecular mass prediction, the purified proteins were applied onto a Superdex 200 increase 10/300 GL (Cytiva) column. Elution was carried out using 10 mM Tris-HCl buffer (pH 8.0) containing 150 mM NaCl. Thyroglobulin (669 kDa), ferritin (440 kDa), aldolase (158 kDa), and conalbumin (75 kDa) were used as molecular mass standards.

### Enzyme assays

Optimal pHs for BbhIV and BbhV were determined using a McIlvaine buffer system (50 mM) containing 1 mM GlcNAcβ-*p*NP as a substrate. After quenching the reaction by adding 1 M Na_2_CO_3_ (10 times the volume), the released *p*-nitrophenolate was quantified by measuring absorbance at 405 nm. Substrate specificity was examined using 4 mM substrates in 50 mM McIlvaine buffer with appropriate pHs (pH 5.0 for BbhIV, pH 5.5 for BbhV, and pH 6.0 for BbhI). The reaction mixtures were incubated at 37 °C in the presence and absence of 1 μM of the enzyme for the indicated time periods, and the reaction was stopped by heating at 80 °C for 10 min. Aliquots were then analyzed by thin layer chromatography (Silica Gel 60, Sigma-Aldrich) with a solvent system consisting of *n*-butanol/acetic acid/water (2:1:1, by volume). The sugars were visualized using a diphenylamine–aniline–phosphoric acid reagent (59). The initial velocities of the reaction were determined by varying the concentrations of GlcNAcβ-*p*NP (0.1–6.25 mM for BbhIV and 0.1–5.0 mM for BbhV), and the data were curve-fitted to the Michaelis–Menten equation. GraphPad Prism v 8.4.3 (GraphPad Software) was used for the calculation of the kinetic parameters. Activities on mucin *O*-glycan chains were examined using PGM as a substrate. PGM, 100 μg, was incubated in the presence and absence of 1 μg of each of the enzymes for 24 h at 37 °C in a total volume of 20 μl. The reaction mixtures were then subjected to *O*-glycan analysis as described below.

### *O*-Glycan analysis

The method for *O*-glycan analysis was the same as described in our previous studies (19, 60). Briefly, after incubation of intact PGM with the purified enzymes or *B. bifidum* variants, the mucin was precipitated by adding 4 times the volume of acetone on ice. The precipitated glycoprotein was suspended in water, and after lyophilization *O*-linked glycans were released by reductive β-elimination. Then, 1 nmol of LNFP I was added as an external standard, and the released glycan alditols and the glycan standard were purified and permethylated, which was followed by a phase partition resulting in separation of nonsulfated *O*-glycan alditols in the organic phase from sulfated *O*-glycan alditols in the aqueous phase (19, 61). The permethylated nonsulfated glycan alditols were dissolved in 2,5-dihydroxybenzoic acid matrix solution (10 mg/ml 2,5-dihydroxybenzoic acid in 50% methanol) and subjected to matrix-assisted laser desorption/ionization–time of flight mass spectrometry (MALDI-TOF/MS) and MALDI-TOF/TOF/MS (LIFT) analyses. AutoflexIII (Bruker Daltonics) was used in the positive ion mode and the reflector mode. Shot counts and laser intensity were kept constant to enable comparison between the samples. Glycosyl compositions were estimated based on mass-to-charge (*m*/*z*) values using the ExPASy GlycoMod tool (62). Isotopic ion peaks with the highest intensities were manually selected in the full mass spectra and used for analysis. Variable modification such as underpermethylation was not considered. Also, only the ion peaks whose glycan compositions were predictable by MS/MS analysis in the control samples and whose mean values were estimated to be larger than 10 pmol/100 ug PGM at least in one sample were considered. Isomeric glycan structures were reported based on MS/MS fragmentation patterns in support of the literature (42, 43).

### Construction of *E. coli*–*Bifidobacterium* shuttle vectors

*E. coli*–*Bifidobacterium* shuttle vectors pMSK216 and 219 were newly constructed by modifying pKKT427, a widely used shuttle vector (3.9 kb) (63). pKKT427 has an extra DNA region that is not essential for plasmid replication, and this region can affect transformation efficiency for different *Bifidobacterium* strains because of the presence of strain-dependent restriction-modification systems (63). First, the DNA regions containing pUC replicon, pTB6 replicon, and the Sp resistance gene (Sp^R^) were PCR-amplified from pKKT427 using primer pairs listed in Supporting Information Table S3 and ligated to yield pMSK187 (3.2 kb). During the process, the multicloning site was removed and the *hup* terminator (∼200 bp) downstream of the pTB6 replicon was replaced with a modified *clpP* terminator (31 bp) (64). Then, to increase the versatility of this new shuttle vector, the Sp^R^ gene was replaced with either the Tet resistance (Tet^R^) or Cm resistance (Cm^R^) gene to yield pMSK219 (4.9 kb) or pMSK216 (3.0 kb). The Tet^R^ gene was amplified by PCR from the genome of *B. longum* subsp. *longum* MCC10025 (locus tag number: MCC10025_0910) isolated previously (65), while the Cm^R^ gene was synthesized at Thermo Fisher Scientific (Waltham) in reference to the nucleotide sequence of 1260 to 1969 in pC194 (GenBank accession: V01277.1) (66). The Cm^R^ gene was placed under the control of the glyceraldehyde-3-phosphate dehydrogenase promoter of BL105A_1176 (67), which was obtained by PCR amplification.

### Gene inactivation in *B. bifidum* JCM 1254

Insertional mutagenesis *via* single cross-over homologous recombination was used to inactivate the *bbhI*, *bbhIV*, and/or *bbhV* genes on the genome of *B. bifidum* JCM1254. Two suicide plasmids were constructed for the purpose. One is pMSK151, which only possesses the Sp^R^ gene and pUC replicon, which was generated by self-ligation of a 1.9-kb *Sac*I-digested fragment of pKKT427 (63). The other is pMSK207, which only carries the Cm^R^ gene and pUC replicon, which was generated by self-ligation of a 1.6-kb PCR-amplified fragment amplified from pMSK216 (see above). Mighty Mix DNA ligation kit (TaKaRa Bio) was used for constructing pMSK151. Then, approximately 500-bp internal region of *bbhI*, *bbhIV*, or *bbhV* was PCR-amplified and was inserted into the *Sac*I site of pMSK151 or the *Nsi*I site of pMSK207. The resulting three pMSK151-based suicide plasmids were separately introduced into *B. bifidum* JCM1254 cells by electroporation, and the respective insertional mutants were screened for Sp^R^. A *bbhI*/*bbhIV* double mutant was obtained by integrating the pMSK207-derived Cm^R^ suicide plasmid into the *bbhI* locus of the *bbhIV-*inactivated Sp^R^ mutant. Integration of the suicide plasmid(s) into the target locus(loci) was verified by direct sequencing of the genomic PCR product(s) (Supporting Information Fig. S3). The electrocompetent cells of *B. bifidum* were prepared as described (68) with slight modifications. Specifically, cells were grown to a logarithmic phase (*A*_600_ = 0.4–0.5) in 100 ml MRS medium (BD DifcoTM) supplemented with 0.2 M NaCl and 0.2 M sucrose and harvested by centrifugation (16,000*g*, 10 min, 4 °C). The cell pellet was then suspended with 20 ml of ice-cold transformation buffer (0.5 M sucrose and 1 mM ammonium citrate [pH 6.0]) and harvested by centrifugation. This washing process was repeated 5 times using 4 ml of the same buffer. Finally, the cells were concentrated in 1 ml of the transformation buffer. Electroporation and subsequent recovery culture were performed as described (69). The plates were anoxically incubated until colonies were visible (typically 2–3 days).

### HPAEC-PAD analysis of monosaccharides released from PGM incubated with *B. bifidum* cells

Monosaccharides that were released from PGM *O*-glycans upon incubation with de-energized cells of *B. bifidum* strains (WT, BbhI^−^, BbhIV^−^, BbhV^−^, and BbhI^−^/BbhIV^−^) were quantified by HPAEC-PAD analysis. The strains were first cultivated in mMRS medium supplemented with mucin *O*-glycan-constituent sugars (Fuc, Gal, GlcNAc, GalNAc, and NeuAc: 0.2% each) to the logarithmic phase (*A*_600_ = 0.4–0.6). Then, the cells were harvested by centrifugation, washed twice with PBS, and resuspended to obtain an *A*_600_ value of 0.8 in PBS containing 5 mM verapamil and 50 μM carbonyl cyanide *m*-chlorophenylhydrazone, which serve as inhibitors of ABC transporters and major facilitator superfamily transporters, respectively (46). The cell suspensions were incubated under anoxic conditions with the inhibitors for 30 min at 37 °C, to which equal volumes of 1% PGM solution were added to initiate the reaction. Samples were taken at the indicated time points and immediately heated at 80 °C for 3 min to cease the reaction. A Dionex ICS-3000 system (Thermo Fisher Scientific) equipped with a CarboPac PA1 column (2 × 250 mm, Dionex) kept at 30 °C was used for sugar quantification. Elution was carried out at a flow rate of 0.25 ml/min with a solvent system of water (mobile phase A), 250 mM NaOH (mobile phase B), and 1 M CH_3_COONa (mobile phase C). The elution scheme (% of mobile phase A/B/C) comprises 94/5.5/0.5 for the first 17 min to detect neutral sugars, followed by a gradient to 46/50/4.0 for the next 10 min to detect NeuAc. The column was washed with 35/50/15 for 10 min after each run. The standard curves were created with known concentrations of sugars.

### Cocultivation of *B. breve* with *B. bifidum* WT or BbhI^−^/BbhIV^−^ strain in PGM-supplemented medium

*B. breve* JCM 1192 carrying pMSK219 (Tet^R^) was cocultivated with *B. bifidum* WT or BbhI^−^/BbhIV^−^ strain in the mMRS medium supplemented with 0.5% PGM and 10 μg/ml Tet. Incubation was initiated by adding *B. breve* cells equivalent to an *A*_600_ value of 0.1 to the medium containing *B. bifidum* cells that correspond to an *A*_600_ value of 0.01. Growth of *B. breve* was measured by CFU on Tet-supplemented GAM agar plates. Note that spontaneous Tet^R^ mutants did not appear when *B. bifidum* alone was incubated in the same medium. Monosaccharides in the supernatant were quantified with HPAEC-PAD under the same conditions as described above.

### Phylogenetic tree construction

The amino acid sequences of the catalytic domains of BbhIV and BbhV were aligned with those of 14 characterized GH84 enzymes listed in the CAZy database as of September 5, 2022 (38), 2 characterized enzymes from *C. perfringens* (34), and 3 uncharacterized homologs from selected mucinolytic gut microbes, *i.e.*, *A. muciniphila* ATCC BAA-835, *Bacteroides caccae* ATCC 43185, and *Bacteroides fragilis* NCTC 9343. The accession numbers of these GH84 homologs are shown in the legend of Figure 7. The catalytic domains were identified by the Protein Families Database (Pfam) (70). Alignment and phylogenetic tree construction were carried out by using Clustal omega (71) and FigTree v1.4.4 (http://tree.bio.ed.ac.uk/), respectively, with default settings.

## Data availability

All data necessary to evaluate the conclusions are presented in this paper. Source data are provided as Supporting Information Tables.

## Supporting information

This article contains supporting information (72).

## Conflict of interest

T. O. is an employee of Morinaga Milk Industry Co, Ltd. Employment of M.S. at Kyoto University is in part supported by Morinaga Milk Industry Co, Ltd.
