# Supplementary material for: GH20 and GH84 β-N-acetylglucosaminidases with different linkage specificities underpin mucin O-glycan breakdown capability of Bifidobacterium bifidum
Source: J Biol Chem. 2023 May 3;299(6):104781. doi: 10.1016/j.jbc.2023.104781 (PMC10245121; doi:10.1016/j.jbc.2023.104781)
Supplement: Supporting information Figures [file mmc2.pdf]

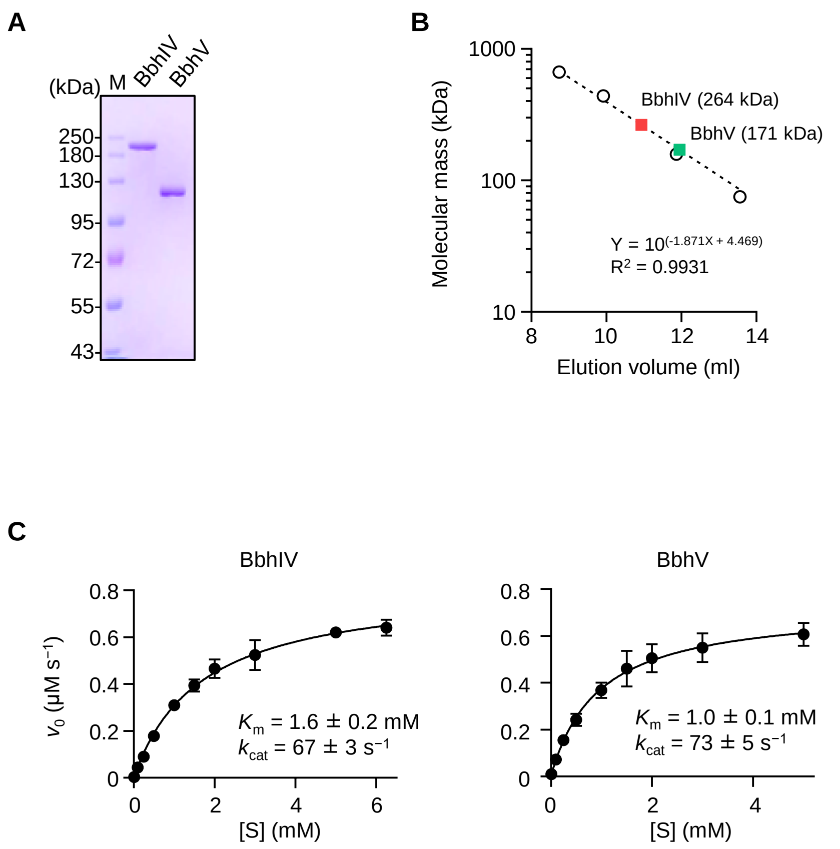

Supporting Information Fig. S1. Characterization of recombinant BbhIV and BbhV from *B. bifidum*. (**A**) The results of SDS-PAGE of purified recombinant BbhIV and BbhV proteins. One μg of each protein was loaded on a 7.5 % acrylamide gel. (**B**) Size-exclusion chromatography of BbhIV and BbhV for molecular size estimation. Thyroglobulin (669 kDa), ferritin (440 kDa), aldolase (158 kDa), and conalbumin (75 kDa) were used as molecular standards. (**C**) S*v*-plots of the hydrolysis of *p*NP-β-GlcNAc by BbhIV (left) and BbhV (right). Data are the mean ± SD of three independent experiments. The kinetic parameters were calculated by curve-fitting the data to the Michaelis-Menten equation.

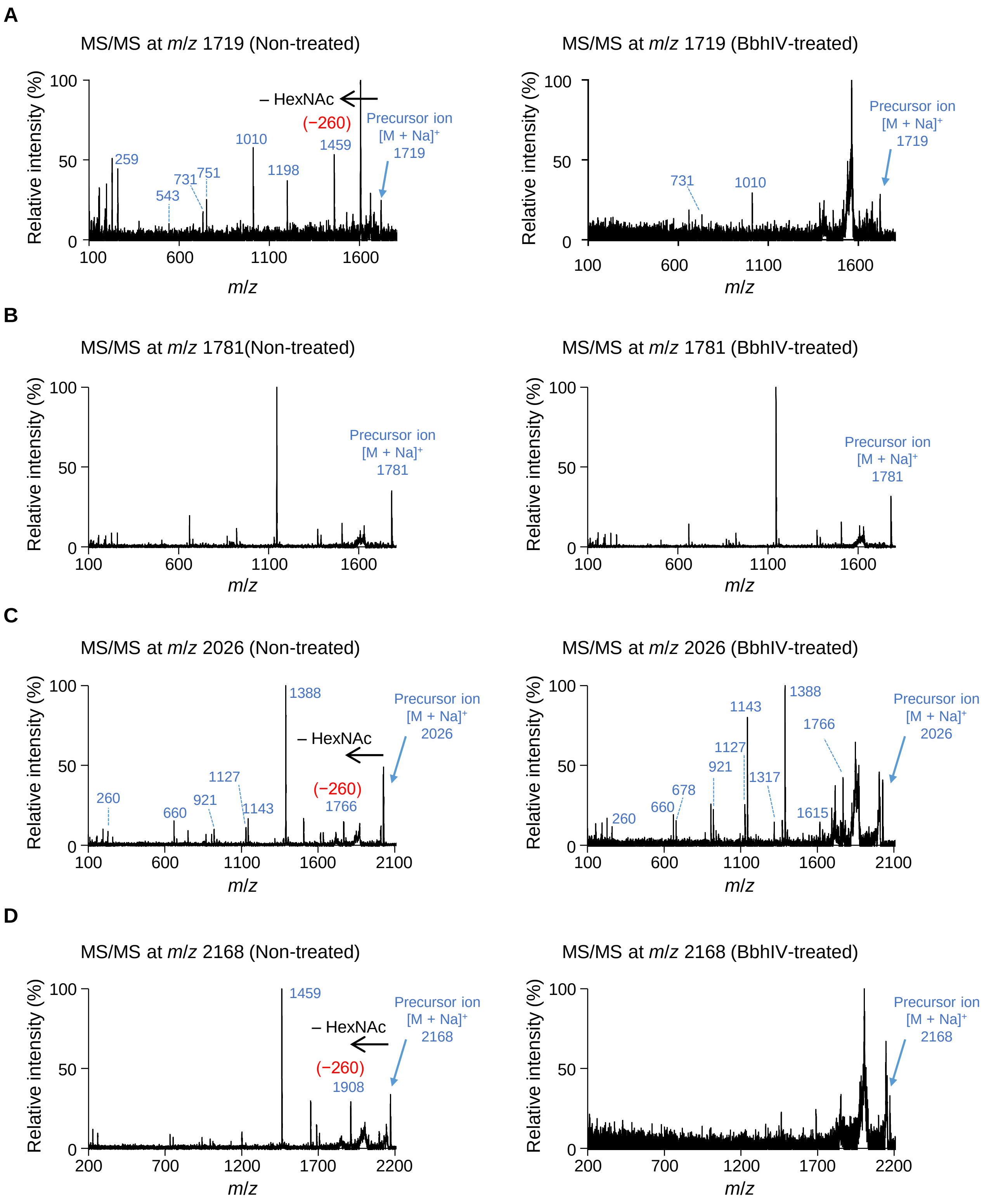

Supporting Information Fig. S2. MS/MS spectra of the PGM *O*-glycan peaks whose estimated amounts were significantly changed upon BbhIV treatment. Representative MS/MS spectra of *m/z* 1719 (**A**), 1781 (**B**), 2026 (**C**), and 2168 (**D**) peaks obtained from the non-treated samples (left) and BbhIV-treated samples (right). Fragmentation of a precursor ion at the site with a terminal HexNAc linkage results in the decrease of 260 Da in MS.

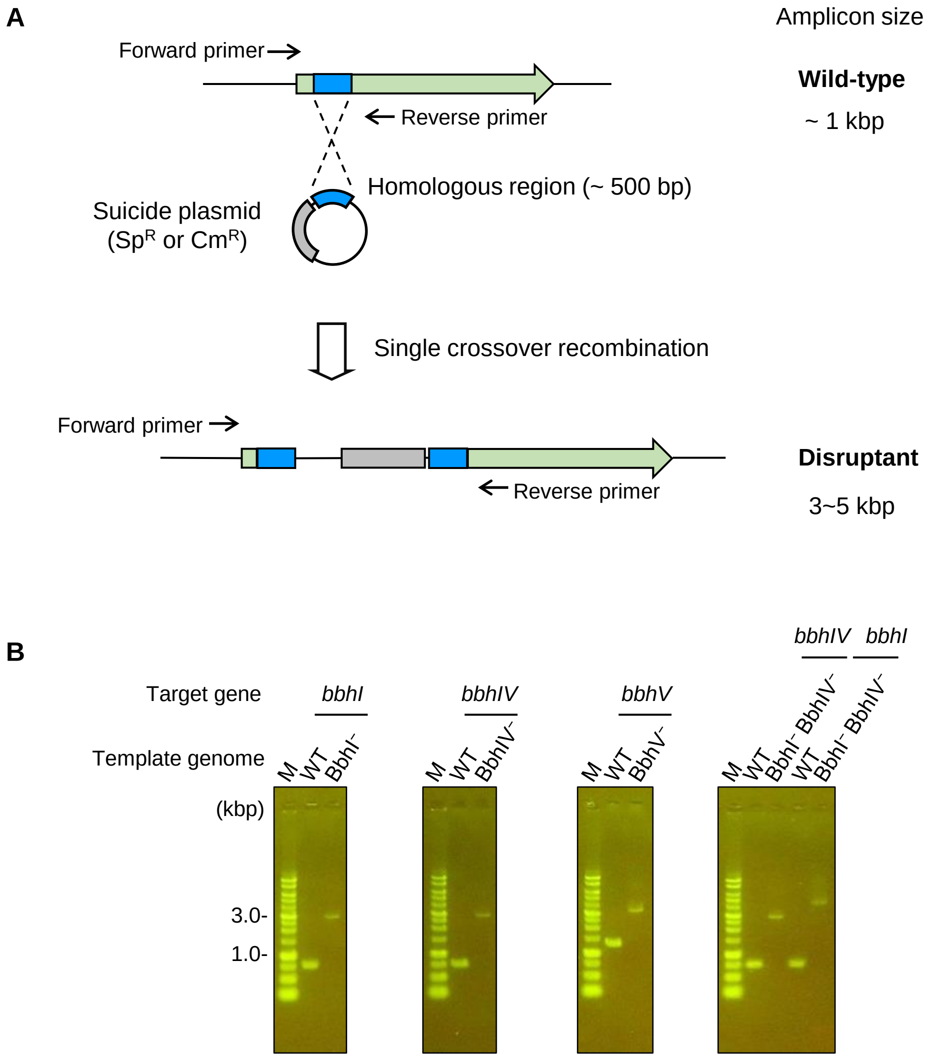

Supporting Information Fig. S3. Disruption of the *bbhI*, *bbhIV*, and/or *bbhV* genes in the genome of *B. bifidum* JCM 1254. (**A**) Schematic representation of a single crossover recombination event for targeted gene inactivation. The homologous regions (~ 500 bp) of *bbhI*, *bbhIV*, and *bbhV*, which were amplified from the genome, were inserted into a suicide plasmid [pMSK151 (Sp^R) or pMSK207 (Cm^R)] (see **Supporting Information Table S3**), and the resulting plasmids were introduced into *B. bifidum* by electroporation for targeted gene disruption. (**B**) The results of agarose-gel electrophoresis of the genomic PCR products. The genomes of WT, $BbhI^-$, $BbhIV^-$, $BbhV^-$, and/or $BbhI^-$ $BbhIV^-$ strains were used as the templates. The primers used were designed to anneal to the positions outside of the regions used for recombination events.

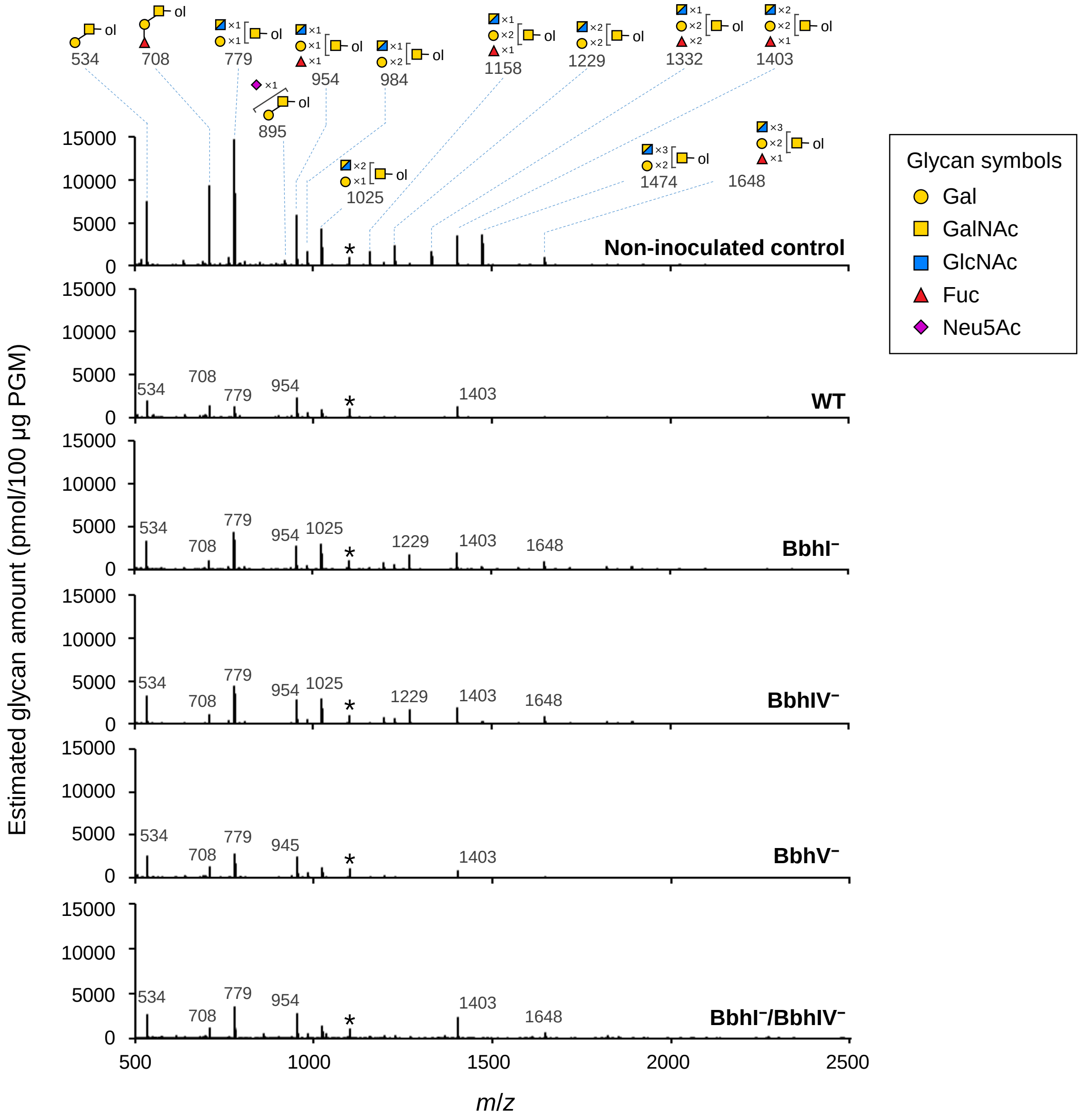

Supporting Information Fig. S4. Full MS spectra of permethylated non-sulfated *O*-glycan alditols obtained from PGM incubated with *B. bifidum* variants. Representative MS profiles of *O*-glycan alditols (*m/z* 500–3000) obtained from PGM incubated with *B. bifidum* WT, BbhI$^-$, BbhIV$^-$, BbhV$^-$, and BbhI$^-$ BbhIV$^-$ strains for 24 h. The MS spectrum obtained from non-inoculated control was also shown. Asterisks are the peak of lacto-*N*-fucopentaose I (LNFP I, *m/z* 1100.6 [M + Na]$^+$) added as an external control for the semi-quantification. See also **Supporting Information Table S2**. Glycan symbols are depicted according to the nomenclature committee of the Consortium for Functional Glycomics (73).

	WT	BbhI⁻	BbhIV⁻	BbhV⁻	BbhI⁻BbhIV⁻
**WT**		0.1174	0.0025	0.9954	0.0027
**BbhI⁻**			0.1503	0.0668	0.1641
**BbhIV⁻**				0.0015	>0.9999
**BbhV⁻**					0.0016
**BbhI⁻BbhIV⁻**					

Supporting Information Fig. S5. *P* values of Tukey's multiple comparison test following one-way ANOVA in Fig. 3C. Estimated total amounts of *O*-glycans obtained from PGM post-cultivation with *B. bifidum* variants were used for comparison, and *P* values obtained are shown. (See **Supporting Information Table S2** for glycan composition and semi-quantification). Data obtained in three independent experiments were used for the analysis.

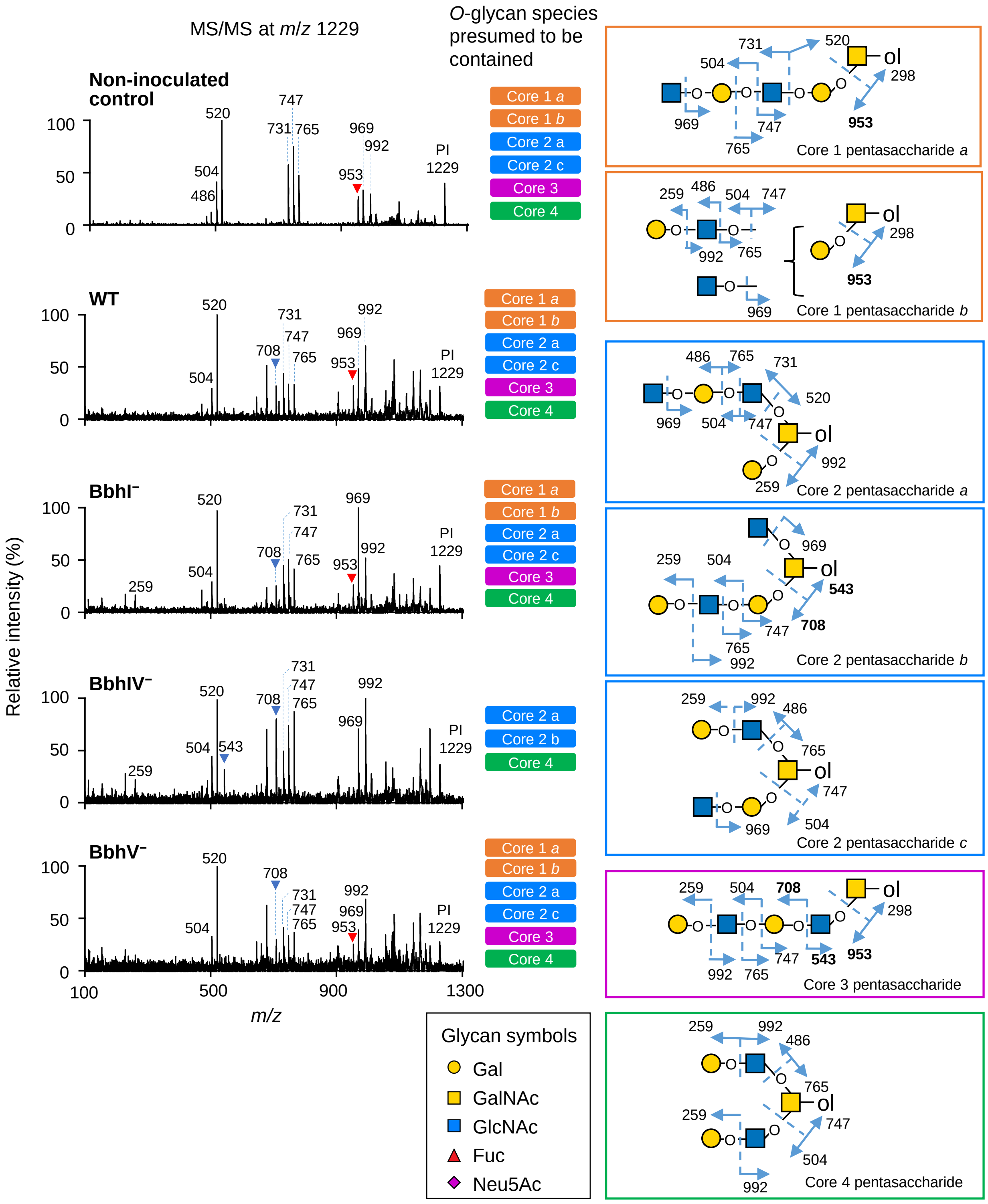

Supporting Information Fig. S6. Different fragmentation patterns observed for the *m/z* 1229 peaks obtained from PGM incubated with *B. bifidum* variants. MS/MS spectra of the *m/z* 1229 peaks (PI: precursor ion) obtained in *O*-glycomic analysis of PGM incubated with *B. bifidum* variants (see **Fig. 3D**) Isomeric pentasaccharide structures predicted from MS/MS fragmentation patterns are shown in the right panels with different core structures boxed with different colors. *O*-glycan species presumed to be contained in the precursor ions are shown in the right of MS/MS spectra. The Glycan symbols are depicted according to the nomenclature committee of the Consortium for Functional Glycomics (73).
